# The Role of the Immunoglobulin Superfamily of Cell Adhesion Molecules in Intellectual Disability

**DOI:** 10.3390/cells15191801

**Published:** 2026-10-02

**Authors:** Nishat Zaman, Karina Bui, Vladimir Sytnyk

**Affiliations:** School of Biotechnology and Biomolecular Sciences, The University of New South Wales, Sydney, NSW 2052, Australia; nishat.zaman@unsw.edu.au (N.Z.); karina.bui@student.unsw.edu.au (K.B.)

**Keywords:** cell adhesion molecule, immunoglobulin superfamily, synapse formation, intellectual disability, Fragile X syndrome, Rett syndrome, Down syndrome, autism spectrum disorder, L1 syndrome, fetal alcohol spectrum disorders

## Abstract

Immunoglobulin superfamily (IgSF) cell adhesion molecules (CAMs) are cell surface glycoproteins that play critical roles during multiple stages of synapse formation, including axon and dendrite growth and guidance, target recognition, axo-dendritic contact formation and its maturation into a functional synapse. Intellectual disability is a condition characterized by significant limitations in intellectual functioning and adaptive behaviors. Growing evidence indicates that deficits in synapse formation underlie the pathogenesis of intellectual disability. In this review, we discuss studies showing that levels and functions of IgSF CAMs are affected in various syndromes and disorders associated with intellectual disability, including Fragile X syndrome, Rett syndrome, Down syndrome, autism spectrum disorder, L1 syndrome and fetal alcohol spectrum disorders. We highlight functional studies in cellular and animal models showing that changes in levels and functions of IgSF CAMs impair synapse formation and induce learning and behavioral abnormalities resembling those found in people with intellectual disability. Together, these studies indicate that IgSF CAMs need to be considered as important therapeutic targets for treating intellectual disability and their functions need to be assessed when new treatments of this condition are developed.

## 1. The Immunoglobulin Superfamily of Cell Adhesion Molecules

Cell adhesion molecules (CAMs) are cell surface glycoproteins that mediate adhesion between adjacent cells and between cells and the extracellular matrix. In addition, CAMs initiate numerous intracellular signaling cascades through binding to their extracellular ligands [1,2,3,4]. Cells of the nervous system express multiple families of CAMs including the cadherin superfamily, integrins, the immunoglobulin superfamily (IgSF), and also molecules that do not belong to the major superfamilies such as neurexins and neuroligins [5,6]. CAMs of all these families were implicated in regulating different aspects of neuronal development and function, and the reader is directed to the recent reviews on this topic for further details on the various functions of cadherins [7,8,9], integrins [10,11,12], neurexins [13,14,15] and neuroligins [16,17]. IgSF members, which are the focus of this review, are briefly overviewed below.

The IgSF is one of the most diverse and oldest families of CAMs [18]. Highly expressed in the developing nervous system, IgSF CAMs are characterized by the presence of one or more immunoglobulin (Ig)-like domains in their extracellular domain [2,18]. The majority of IgSF CAMs are type I transmembrane proteins [19]. However, some IgSF CAMs are attached to the cell surface plasma membrane via a glycosylphosphatidylinositol (GPI) anchor [20]. IgSF CAMs mediate calcium-independent cell adhesion through their extracellular Ig-like domains, which typically interact homophilically with Ig-like domains of identical CAMs on adjacent cell membranes [2,19,21]. However, IgSF CAMs also facilitate cell adhesion via heterophilic interactions with other CAMs and extracellular matrix proteins [22,23,24,25]. Along with Ig-like domains, the extracellular domains of IgSF CAMs often include other protein modules, with fibronectin type III (FNIII) repeats being the most common [18].

**Figure 1:**
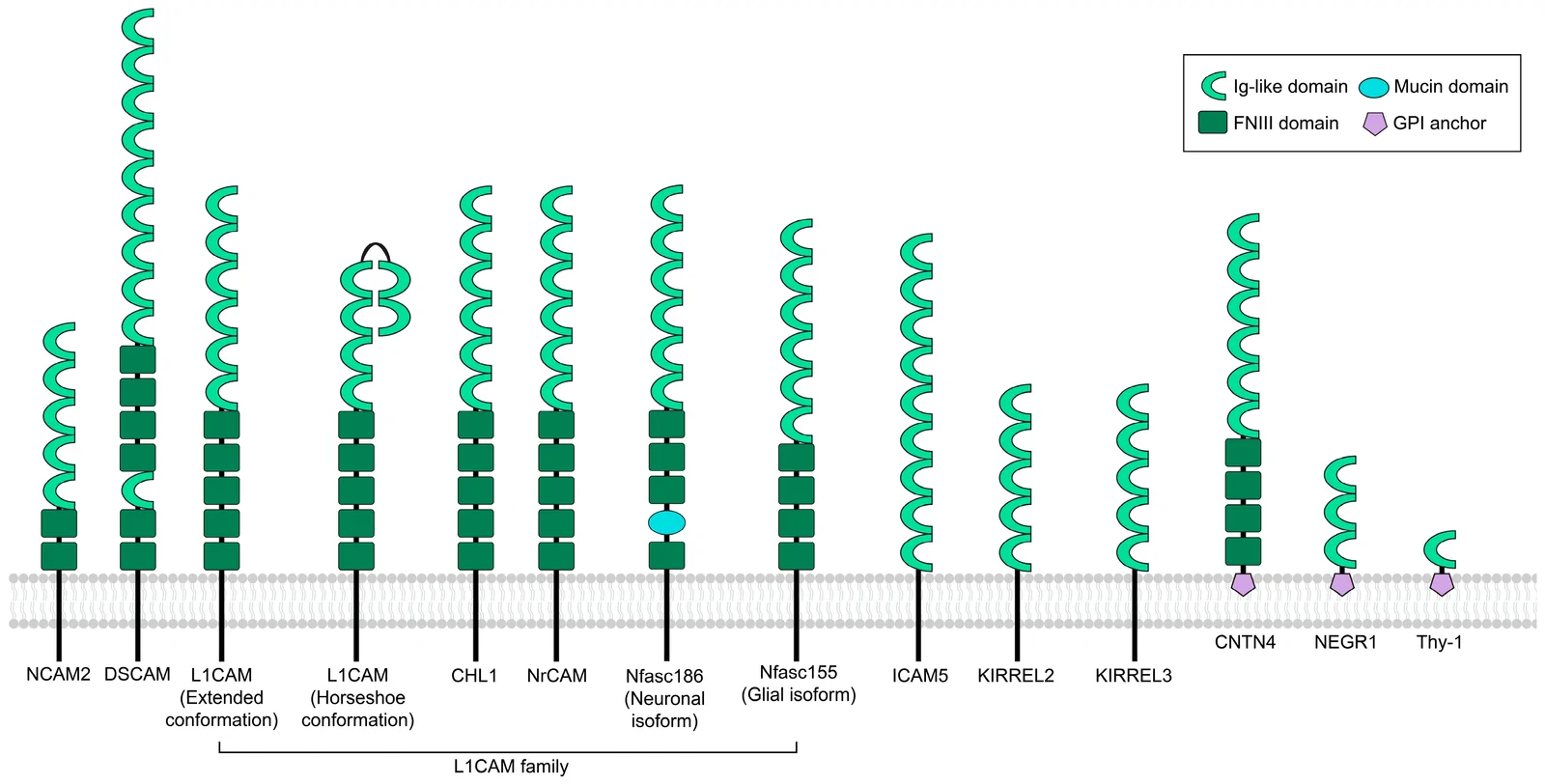
Examples of IgSF CAMs involved in intellectual disability. IgSF CAMs contain one or more immunoglobulin (Ig)-like repeats in their extracellular domain and can contain additional modules, most commonly fibronectin type III (FNIII) modules. Based on their extracellular domain composition, IgSF CAMs are divided into distinct subfamilies. An example of such a subfamily is the L1CAM family, which consists of L1CAM, CHL1, NrCAM and neurofascin. The extracellular domain of L1CAM family members consists of 6 Ig-like and 5 FNIII modules and was proposed to adopt either an extended conformation or folded, horseshoe conformation (shown only for L1CAM), which supports L1CAM-mediated cell adhesion. Alternative splicing generates additional isoforms of individual members, such as neuronal Nfasc186 and glial Nfasc155 isoforms of neurofascin. While most IgSF CAMs are transmembrane proteins, some CAMs, including CNTN4, NEGR1 and Thy-1, are attached to the plasma membrane via a GPI anchor.

Based on their domain composition, IgSF CAMs can be divided into multiple, distinct subfamilies [18,26]. Prominent subfamilies of IgSF CAMs include the neural cell adhesion molecule (NCAM), L1CAM, IgLON, contactin, nectin and sidekick subfamilies [20,24,27,28,29,30,31] (Figure 1). Accumulating evidence indicates that multiple members of the IgSF are implicated in various syndromes associated with intellectual disability, which are briefly introduced below.

## 2. Intellectual Disability

Intellectual disability (ID) is a neurodevelopmental disorder characterized by significant limitations in intellectual functioning (intelligence quotient (IQ) < 70) and adaptive behaviors with an onset before the age of 18 [32,33]. For most individuals, ID is diagnosed in early childhood due to developmental delays [34]. In clinical practice, the diagnosis of ID in a person is based on an IQ below 70 and deficits in two or more adaptive behaviors, such as personal care, social skills and communication [35]. The term global developmental delay (GDD) is commonly used for young children under the age of 5 [36]. GDD refers to a child having significant delays in two or more developmental domains, including speech and language, gross/fine motor, cognition, social/personal, and daily activities. The term ID is generally applied to children aged 5 years and above [35]. Many children with GDD are diagnosed with ID later in life when intellectual functioning can be reliably assessed using standardized tests [32,37,38].

ID affects approximately 1–3% of the global population and is one of the top ten causes of disease burden worldwide [33,39,40,41]. Currently, there are no pharmacological therapies available for ID [42]. ID is an important healthcare issue as it is a lifelong condition, has limited treatments and poses substantial economic costs to families and society [43,44,45]. ID is classified into four categories of severity: mild, moderate, severe, and profound [32]. The severity of ID is ascertained based on a person’s level of adaptive functioning as this determines the amount of support they will require. The majority (around 85%) of ID cases can be attributed to mild forms of ID [33]. ID can appear as an isolated phenotype (non-syndromic ID) or be associated with other clinical features (e.g., physical deformities or metabolic defects) as part of a syndrome (syndromic ID) [35,44,46]. Common syndromic forms of ID include Down syndrome, Fragile X syndrome and Rett syndrome [47]. Individuals with ID frequently present other comorbidities including autism, epilepsy and sensory impairments [34,48].

ID can be caused by various environmental and/or genetic factors that affect the development and functioning of the central nervous system during the prenatal, perinatal or postnatal period [49,50]. Environmental factors such as infections, maternal exposure to toxins during pregnancy, premature birth and malnutrition can cause ID [34,49]. It is well established that ID has a strong genetic component, and most severe forms of ID have a genetic basis [46,51,52,53]. Overall, 25–50% of ID cases are due to genetic causes, though this number increases with ID severity [54].

Genetic causes of ID include copy number variations (CNVs), single-nucleotide variants (SNVs), chromosomal rearrangements, and aneuploidies [40,46]. Down syndrome is the most common genetic form of ID, and is caused by the partial or total trisomy of human chromosome 21 [55,56]. The next leading genetic cause of ID is Fragile X syndrome, which is caused by mutations in the *FMR1* gene located on the X chromosome [57]. In addition, hundreds of rare variants have been identified as genetic causes of ID [40,58]. These variants are both inherited and de novo mutations [59]. De novo SNVs and CNVs are a major cause of moderate and severe cases of ID [52,60]. Advances in next-generation sequencing (NGS) technologies have facilitated the rapid identification of ID candidate genes over the past decade [34,48]. To date, more than 1000 genes have been implicated in ID [43]. ID is the most frequent reason for referral to genetic services [34].

However, finding ID-causing mutations in candidate genes is challenging due to the extreme genetic heterogeneity and rarity of mutations in any given gene in the ID population [61]. Due to this, large numbers of patients are needed for the detection of recurrently mutated genes [48]. Up to 50% of ID patients remain without a genetic diagnosis [62]. The identification of a genetic etiology is essential for the development of personalized treatments, and for furthering our knowledge of the pathogenesis of ID [63].

Synapses are the fundamental information processing units of the brain, and changes in synaptic strength underlie learning and memory [64,65]. Synaptic dysfunction arising from perturbations in synapse structure and function is the basis of many brain disorders, which are termed “synaptopathies” [65,66,67]. Despite the immense genetic heterogeneity of ID, many ID genes converge onto common molecular and cellular pathways [33,40]. Network-based analyses have revealed that a substantial proportion of ID genes encode synaptic proteins involved in synapse formation and maturation [43,68,69,70,71,72].

A growing body of evidence indicates that defects in synapse formation underlie the pathogenesis of ID. The first indication that impaired synaptogenesis is a major cause of ID came from the analysis of human post-mortem brain tissue. A common neuropathological change associated with ID is a change in the density and/or morphology of dendritic spines [73,74]. Since the 1970s, studies have shown that cortical and hippocampal neurons from patients with ID have reduced numbers of dendritic spines compared to unaffected individuals [74,75,76,77,78,79]. In addition, ID patients show an increased prevalence of long, thin spines, which are characteristic of immature synapses [77,78,79,80,81,82]. Consistent with these findings, neurons from human and mouse models of ID show impairments in excitatory synapse formation and/or maturation [83,84,85,86,87,88,89,90]. Normal brain function also requires the proper balance between excitation and inhibition within neural circuits [91,92]. Disruptions in the excitatory/inhibitory (E/I) balance are hypothesized to underlie the cognitive deficits observed in ID and other neurodevelopmental disorders [93,94,95].

Given the important roles that IgSF CAMs play in synapse formation and maturation [2,20,96], it is not surprising that IgSF CAMs have been implicated in intellectual disability [97,98,99,100,101,102,103]. We systematically searched for literature in Google Scholar and PubMed indicating that IgSF CAMs are involved in genetic syndromes that are known to cause intellectual disability, including Fragile X syndrome, Rett syndrome, and Down syndrome. We also review studies highlighting a role for IgSF CAMs in autism spectrum disorder and fetal alcohol spectrum disorders, since intellectual disability is a feature of both of these conditions. Finally, we also discuss L1 syndrome as a condition associated with intellectual disability caused by mutations in an IgSF CAM.

## 3. Fragile X Syndrome and IgSF CAMs

Fragile X syndrome (FXS) is a neurodevelopmental disorder that is the most common inherited form of ID and the leading monogenic cause of autism spectrum disorder [104,105,106]. The estimated prevalence of FXS is 1:7143 in males and 1:11,111 in females [107]. Clinical features of this syndrome include language delays, poor attention, hyperactivity, anxiety, seizures, impulsivity and aggression [108]. In the United States, the mean age of FXS diagnosis is 36 months [109]. There are currently no approved therapies for FXS [110].

FXS is caused by the expansion of CGG trinucleotide repeats (≥200) in the 5′ untranslated region (5′ UTR) of the fragile X messenger ribonucleoprotein 1 (*FMR1*) gene [111,112,113]. The normal *FMR1* allele consists of 5–44 CGG repeats [107]. The expanded *FMR1* allele, consisting of more than 200 CGG repeats, is known as the full mutation. This expansion mutation results in the methylation of the CGG repeat and promoter CpG island of the *FMR1* gene, leading to heterochromatin formation and silencing of *FMR1* expression [113,114,115]. Consequently, this leads to reduced production or complete loss of the protein encoded by the *FMR1* gene—fragile X messenger ribonucleoprotein (FMRP). Since FXS is X-linked (the *FMR1* gene is located on chromosome Xq27.3), females are less severely affected compared to males, due to compensation by the unaffected X chromosome [107,108,116].

FMRP is an RNA-binding protein that regulates translation [113,117]. FMRP is expressed in many tissues and is enriched in the brain [118,119]. In the brain, FMRP is expressed in both neurons and glial cells [120,121]. FMRP represses the translation of specific mRNAs, many of which encode proteins that are important for synaptic development and plasticity [117], including postsynaptic density protein-95 (PSD-95) [122], the NMDA receptor subunit NR2A (GluN2A) [123], amyloid precursor protein (APP) [124], microtubule-associated protein 1B (MAP1B) [125] and the voltage-gated potassium channel Kv4.2 [126].

FMRP possesses three RNA-binding motifs, including two heterogeneous nuclear RNP K homology (KH) domains and an RGG box [127]. In addition, FMRP contains nuclear localization and export signals, and promotes the nuclear export of N^6^-methyladenosine (m^6^A)-modified mRNA transcripts [128]. Furthermore, FMRP also promotes the trafficking of mRNA granules along neuronal dendrites, which is essential for local protein synthesis that occurs in response to synaptic stimulation [129,130,131]. FMRP represses translation through multiple mechanisms [117]. FMRP associates with polyribosomes [132,133,134], and inhibits translation elongation through stalling ribosome translocation [135,136,137,138]. Additionally, FMRP represses translation through interacting with microRNAs (miRNAs) and components of the RNA-induced silencing complex (RISC) [122,123,139,140,141]. It has also been reported that FMRP prevents the formation of the translation initiation complex [142,143].

The loss of FMRP in FXS leads to dysregulated translation [117]. Studies have shown that basal protein synthesis is elevated in the brains of *Fmr1* knockout mice [144,145,146]. In addition, de novo protein synthesis is elevated in fibroblasts and lymphoblastoid cells from FXS patients [147,148], and in FMRP-deficient human neurons [149]. Further, the loss of FMRP in mouse neurons impairs the BDNF-induced local protein synthesis of PSD-95 in dendrites, which is crucial for long-term potentiation (LTP) [150]. This defect in local PSD-95 synthesis in FMRP-deficient neurons was rescued through inhibiting the mTORC1-S6K1 pathway. Indeed, studies have shown that the aberrant translation observed in FXS models is due to the hyperactivation of multiple signaling pathways including the PI3K-Akt-mTOR and the mGluR5-ERK1/2 cascades [146,151,152,153]. These signaling pathways activate the protein synthesis machinery through promoting the assembly of the translation initiation complex [151,154,155].

A key neuropathological feature of FXS is impaired synapse maturation. An increased density of immature dendritic spines with thin, elongated morphology has been observed in the brains of both *Fmr1* knockout mice and FXS patients [156,157,158,159]. A recent report showed that cultured hippocampal neurons from *Fmr1* knockout mice exhibit reduced dendritic growth and branching, increased soma size, and upregulated cell surface levels of GluA2 and GluN2A along dendrites [160]. Additionally, excitatory synapses of *Fmr1* knockout mice have increased surface levels of GluN2A, whereas the surface levels of GluN2B are reduced.

Furthermore, *Fmr1* knockout mice have impaired LTP in various brain regions [161,162,163,164]. In addition, metabotropic glutamate receptor-dependent long-term depression (mGluR-LTD) is enhanced in the hippocampus [165,166]. Long-term synaptic plasticity is essential for learning and memory [167,168]. Behavioral analyses have demonstrated that *Fmr1* knockout mice have cognitive impairments, including deficits in cognitive flexibility [169], trace fear memory [161], episodic memory [170], and object novelty detection [171].

The dysregulation of protein synthesis caused by the loss of FMRP is considered to underlie the neuronal and synaptic deficits observed in FXS [144,148,149,165,172,173]. FMRP has hundreds of mRNA targets in human forebrain organoids and in the mouse brain [135,174]. A subset of these mRNA targets encodes CAMs, and functional studies have demonstrated that FMRP represses the translation of specific IgSF CAMs, including intercellular adhesion molecule 5 (ICAM5) [175] and Down syndrome cell adhesion molecule (DSCAM) [176,177]. Below, we discuss the association between FXS and ICAM5, DSCAM, and other IgSF members.

### 3.1. ICAM5 in FXS

ICAM5 is an IgSF member containing 9 Ig-like domains in its extracellular domain (Figure 1). It is exclusively expressed in the telencephalon of the mammalian brain, and is localized on the somatodendritic membranes of neurons, but is not present on axons [178,179]. ICAM5 negatively regulates dendritic spine maturation and promotes the formation of dendritic filopodia, which are the precursors of mature, mushroom-shaped spines [180,181,182]. ICAM5 is highly expressed in dendritic filopodia [180]. As filopodia transition into mature spines, the levels of ICAM5 decrease. Importantly, the activation of NMDA receptors increases the activity of matrix metalloproteinases 2 and 9 (MMP-2 and MMP-9), which cleave and release the ICAM5 ectodomain from dendritic filopodia, resulting in spine maturation [181].

Reports have highlighted an association between ICAM5 and FXS. FMRP directly binds to and represses the translation of *ICAM5* mRNA transcripts [175]. In cultured wild-type mouse cortical neurons, the shRNA knockdown of FMRP expression led to an increase in ICAM5 protein levels, whereas *ICAM5* mRNA levels were unaffected. In addition, the shRNA-mediated suppression of FMRP expression in wild-type neurons resulted in elevated levels of ribosome-bound *ICAM5* mRNA transcripts.

Furthermore, ICAM5 protein levels were increased in the hippocampus, prefrontal cortex and amygdala of 3-week-old *Fmr1* knockout versus wild-type mice (Table 1). However, *ICAM5* mRNA levels were similar in these brain regions in 3-week-old wild-type and *Fmr1* knockout mice, indicating that in *Fmr1* knockout mice, *ICAM5* mRNA translation is elevated (Table 1). Supporting this idea, the levels of ribosome-bound *ICAM5* mRNA transcripts were increased in the hippocampus of *Fmr1* knockout mice compared to wild-type mice.

Moreover, dendritic spine pruning and maturation were impaired in the hippocampus of *Fmr1* knockout mice. In Golgi-stained hippocampal neurons from 3-week-old *Fmr1* knockout mice, the density of dendritic spines was increased compared to wild-type neurons. Also, in *Fmr1* knockout mice, the percentage of filopodia-like, immature spines was increased, whereas the percentage of mature, mushroom-shaped spines was reduced. Importantly, shRNA-induced ICAM5 knockdown in cultured *Fmr1* knockout neurons rescued the deficits in dendritic spine maturation and promoted an increase in the percentage of mature, mushroom-shaped spines.

Lastly, *Fmr1* knockout mice exhibit various cognitive and behavioral deficits. The shRNA-mediated downregulation of ICAM5 expression in the hippocampal dentate gyrus of *Fmr1* knockout mice ameliorated impairments in spatial and fear memory, social interaction, and exploratory and anxiety-like behaviors. Interestingly, the accumulation of ICAM5 in the postsynaptic membrane is regulated by calsyntenin-1 (CLSTN1) [183].

CLSTN1 is a postsynaptic adhesion molecule belonging to the cadherin superfamily [184,185]. During postnatal development, CLSTN1 transports NMDA receptors to synapses, and promotes dendritic spine maturation [186]. The *CLSTN1* mRNA transcript was identified as an FMRP target in human forebrain organoids and in the mouse brain [135,174]. In *Fmr1* knockout mice, CLSTN1 protein levels were reduced in the medial prefrontal cortex during postnatal development, and this reduction correlated with an increase in ICAM5 protein levels. The shRNA-mediated downregulation of CLSTN1 expression resulted in an increase in ICAM5 protein levels in the synaptosomal membranes of cultured wild-type neurons. In addition, overexpressing CLSTN1 in *Fmr1* knockout neurons rescued impairments in dendritic spine maturation.

Collectively, these findings support a role for ICAM5 and CLSTN1 as potential therapeutic targets for treating dendritic spine abnormalities in FXS. It is interesting that the expression of both ICAM5 and CLSTN1 is dysregulated in the brains of *Fmr1* knockout mice. Future studies should focus on uncovering the molecular mechanisms through which CLSTN1 reduces ICAM5 protein levels at the postsynaptic membrane, and also on determining whether the expression of ICAM5 and CLSTN1 is altered in human models of FXS.

### 3.2. DSCAM in FXS

The *DSCAM* mRNA transcript was identified as an FMRP target in human forebrain organoids and in the mouse brain [135,174]. Functional studies using *Drosophila* have shown that FMRP influences multiple aspects of neurodevelopment through regulating Dscam expression. In the *Drosophila* brain, FMRP directly binds to *Dscam* mRNA, and represses Dscam protein expression at the translational level [176,177]. In FMRP-deficient *Drosophila*, Dscam protein levels were increased, whilst *Dscam* mRNA levels were unchanged (Table 1). The elevated Dscam protein levels in FMRP-deficient *Drosophila* resulted in axonal targeting errors of the posterior scutellar mechanosensory neurons. In addition, the aberrations in axonal targeting led to impaired touch perception. Importantly, reducing the levels of Dscam in FMRP-deficient *Drosophila* ameliorated the impairments in axonal targeting.

Furthermore, in *Drosophila*, the size of the presynaptic arbor of somatosensory neurons is controlled by Dscam expression levels [177]. FMRP represses Dscam protein expression to restrict the growth of presynaptic arbors, with FMRP-null mutants having enlarged presynaptic arbors compared to wild-type *Drosophila*. It was shown that Dscam promotes presynaptic arbor development through Abelson tyrosine kinase (Abl kinase) activation [187]. The cytoplasmic domain of Dscam interacts with and activates Abl kinase. Notably, administration of nilotinib, an Abl kinase inhibitor, to FMRP-null mutants rescued the overgrowth of presynaptic arbors.

These findings indicate that Abl kinase may be a potential drug target for ameliorating the effects of upregulated Dscam expression in FXS. However, to date, there are no studies demonstrating that DSCAM expression is dysregulated in mammalian (e.g., mouse and human) models of FXS. Future research should focus on examining whether the functional association between DSCAM and Abl kinase is also affected in mammalian models of FXS.

### 3.3. NCAM2 in FXS

NCAM2 is an IgSF member containing 5 Ig-like and 2 FNIII domains in its extracellular domain (Figure 1). The *NCAM2* mRNA transcript was identified as an FMRP target both in human forebrain organoids and mouse fetal brains [174]. To date, there are no functional studies demonstrating that FMRP regulates the translation of *NCAM2* mRNA. FXS patients exhibit high genetic and phenotypic heterogeneity [188,189]. In a previous report, Susco and colleagues generated isogenic *FMR1*^y/+^ and *FMR1*^y/−^ human pluripotent stem cell (hPSC) lines using CRISPR-Cas9 gene editing to study how the complete loss of FMRP promotes molecular and cellular changes relevant for FXS, independent of genetic background [190]. These hPSC lines were differentiated into excitatory neurons. It was shown that NCAM2 protein levels were increased in isogenic *FMR1*^y/−^ versus *FMR1*^y/+^ hPSC-derived excitatory neurons, suggesting that FMRP represses *NCAM2* mRNA translation (Table 1) [191].

Individuals with FXS and *Fmr1* knockout mice have an increased density of immature, filopodia-like dendritic spines [158,159]. Importantly, the correct dosage of *NCAM2* is critical for proper excitatory synapse maturation [192]. The overexpression of NCAM2 in cortical neurons from the first day in culture results in the impaired maturation of filopodia into stable dendritic spines [192]. However, NCAM2 is crucial for synapse maintenance in mature neurons. In mature cultured hippocampal neurons, NCAM2 is enriched at excitatory synapses and the acute disruption of NCAM2 trans-homophilic interactions promotes the disassembly of excitatory synapses [193].

Environmental enrichment has been shown to improve multiple aspects of brain development, and has been proposed as a therapeutic intervention for neurodevelopmental disorders, including FXS [194,195]. Interestingly, it was found that environmental enrichment led to an upregulation of *NCAM2* mRNA levels in the hippocampus of *Fmr1* knockout mice (Table 1) [194]. In addition, environmental enrichment ameliorated cognitive impairments, stereotyped behaviors and anxiety in *Fmr1* knockout mice.

Together, these studies highlight a potential role for NCAM2 as a therapeutic target for FXS. While NCAM2 protein levels were found to be elevated in isogenic *FMR1*^y/−^ versus *FMR1*^y/+^ hPSC-derived excitatory neurons [191], additional research is required to uncover whether NCAM2 expression is dysregulated in in vivo FXS models, such as *Fmr1* knockout mice. Future work should also focus on investigating whether there is a direct regulatory relationship between NCAM2 and FMRP, and the molecular and cellular mechanisms through which NCAM2 contributes to the phenotypes of FXS.

### 3.4. Thy-1 in FXS

Lipid rafts are specialized microdomains in the plasma membrane that are enriched in cholesterol and glycosphingolipids, and act as platforms for signal transduction through compartmentalizing signaling proteins and lipids [196,197,198]. Proteins are targeted to lipid rafts by lipid modifications such as a glycosylphosphatidylinositol (GPI) anchor [199]. In the brain, lipid rafts play crucial roles in numerous neurodevelopmental processes including neuronal differentiation, synaptogenesis, axon guidance and myelination [196,200]. The composition of lipid rafts is altered in various brain disorders such as Alzheimer’s disease and schizophrenia [196]. Importantly, a subset of the FMRP mRNA targets encode proteins that are involved in lipid synthesis and transport [135,196].

Thy-1 is a GPI-anchored membrane glycoprotein belonging to the IgSF [20,201]. The expression of Thy-1 in the brain is developmentally regulated, with Thy-1 levels strongly increased during early postnatal development [202]. Thy-1 is expressed both in neurons and glial cells [203,204,205,206]. In the brain, Thy-1 is enriched at lipid rafts [199]. Multiple reports have shown that Thy-1 regulates neurite outgrowth [207,208,209,210]. For example, when Thy-1-expressing NG115-401L neuronal cells are grown on a monolayer of astrocytes, neurite outgrowth is inhibited [208]. However, neurite outgrowth is restored when the cells are treated with soluble Thy-1 or antibodies against Thy-1, presumably through blocking the interaction between neuronal Thy-1 and an astrocytic ligand. Other studies found that treating multiple cell types with antibodies against Thy-1 stimulated neurite outgrowth [207,209,210].

Thy-1 is present on synaptic vesicles, and treating PC12 cells with antibodies against Thy-1 inhibits the regulated release of neurotransmitters [211]. Further, Thy-1 mediates the bidirectional communication between neurons and astrocytes [212,213]. Neuronal Thy-1 interacts with astrocytic integrin β1 to suppress the proliferation and activation of astrocytes [212]. In addition, astrocytic αVβ3 integrin binds to neuronal Thy-1 to restrict neurite outgrowth, and promote the retraction of existing processes through inducing the clustering of Thy-1 [213]. Mice that are deficient in Thy-1 have impaired LTP in the dentate gyrus of the hippocampus [214], and are unable to use socially transmitted cues to direct their food preferences [215].

Using iTRAQ proteomics, Kalinowska and colleagues found that the lipid raft proteome is changed in the brains of adult *Fmr1* knockout mice compared to wild-type mice [196]. Interestingly, it was found that the protein abundance of Thy-1 was decreased in lipid rafts from *Fmr1* knockout mice (Table 1). Additionally, using Western blot analysis, it was shown that the protein levels of Thy-1 in cortical, hippocampal and cerebellar lysates were comparable between wild-type and *Fmr1* knockout mice. These findings suggest that a reduction in Thy-1 levels in lipid rafts in *Fmr1* knockout mice is not due to overall changes in protein expression levels, but rather due to other cellular mechanisms.

Although lipid rafts contain numerous proteins encoded by mRNA transcripts that are FMRP targets, there are currently no studies showing that *THY1* mRNA is a target of FMRP [135,174]. Importantly, the GPI anchor and lipid raft localization of Thy-1 are required for Thy-1 to mediate its biological functions [216,217,218]. For example, replacing the GPI anchor of Thy-1 with a polypeptide transmembrane domain alters the membrane localization of the protein, and abolishes the inhibitory effect of Thy-1 on neurite outgrowth when NG115-401L neuronal cells are grown on an astrocytic monolayer [218].

Notably, a subset of the FMRP mRNA targets encode proteins that play roles in lipid homeostasis [135,174,196]. One of these mRNA targets encodes phosphatidylinositol glycan biosynthesis class Q (PIGQ), which is involved in the first step of GPI anchor biosynthesis [135,174,196,219,220]. Hence, defects in GPI anchor biosynthesis were proposed to underlie a reduction in Thy-1 protein abundance in lipid rafts from *Fmr1* knockout mice [196]. In addition, impairments in protein trafficking or membrane anchoring could also contribute to this effect [196,219].

It would be valuable to elucidate the mechanisms through which FMRP deficiency contributes to the reduced abundance of Thy-1 and possibly other GPI-anchored IgSF CAMs in lipid rafts. Given the important role of lipid rafts during brain development [200], increasing our knowledge of the mechanisms through which lipid rafts are altered in FXS may uncover novel molecular pathways for therapeutic interventions.

### 3.5. NrCAM in FXS

In the human brain, the ratio of neurons to glial cells is close to 1:1, with region-specific differences [221]. Astrocytes are the most abundant glial cell type in the brain and regulate many aspects of synapse formation and function [222,223,224]. Protoplasmic astrocytes of the grey matter have highly ramified morphologies and infiltrate the surrounding neuropil [225,226]. These astrocytes possess fine, perisynaptic processes that ensheath neuronal synapses [225,227].

In the brain, tripartite synapses consist of a pre- and postsynaptic compartment that are in direct contact with an astrocytic process [228,229,230]. At tripartite synapses, astrocytes regulate neuronal excitability and synaptic transmission through the release of gliotransmitters [229]. Astrocytes regulate the structure and function of both excitatory and inhibitory synapses [230]. Importantly, studies have indicated that astrocytes are involved in the pathophysiology of FXS, as reviewed in [231]. The proper formation and maturation of synapses requires the expression of FMRP in astrocytes.

The *NRCAM* mRNA transcript was identified as an FMRP target both in human forebrain organoids and mouse fetal brains [174]. NrCAM, an IgSF member containing 6 Ig-like and 5 FNIII domains in its extracellular domain (Figure 1), is expressed in neurons and astrocytes, and is enriched at tripartite synapses in vivo [232,233]. NrCAM regulates astrocyte morphogenesis and inhibitory synapse development [233]. The transcellular, homophilic binding between astrocytic and neuronal NrCAM limits the growth of astrocytic processes into the neuropil in the mouse brain [233].

In addition, astrocytic NrCAM interacts with neuronal NrCAM that is associated with the scaffolding protein gephyrin at inhibitory postsynapses. In the mouse visual cortex, the loss of astrocytic NrCAM, neuronal NrCAM or both astrocytic and neuronal NrCAM markedly reduces inhibitory synapse numbers without affecting excitatory synapse numbers. These findings support a model where astrocytic NrCAM supports inhibitory synapse formation and/or stabilization through trans-interactions with neuronal NrCAM.

Recently, it was reported that 95 proteins were differentially expressed in cortical astrocytes from *Fmr1* knockout mice [234]. Interestingly, protein levels of NrCAM were downregulated in *Fmr1* knockout astrocytes (Table 1). Since astrocytic NrCAM is essential for proper inhibitory synaptic development, a reduction in NrCAM protein levels in *Fmr1* knockout astrocytes could potentially result in alterations in the E/I balance in the *Fmr1* knockout mouse brain. Normal brain function requires the correct balance between excitatory and inhibitory synapses during neural circuit formation [91]. Altered E/I balance is hypothesized to underlie the cognitive deficits observed in ID and other neurodevelopmental disorders [93,94,95].

Dysfunction of the GABAergic system is considered to underlie neural circuit deficits in *Fmr1* knockout mice [234]. For example, *Fmr1* knockout mice have an impaired E/I balance in the sensory neocortex, leading to the hyperexcitability of neocortical circuits [235]. In addition, the inhibitory synapse density was reduced in the auditory cortex of *Fmr1* knockout mice, whereas the excitatory synapse density was unaltered [234]. Furthermore, both male NrCAM-null and *Fmr1* knockout mice exhibit deficits in social interaction [236,237].

Further research is required to elucidate the mechanisms through which NrCAM protein levels are decreased in *Fmr1* knockout cortical astrocytes. It is currently unclear whether this reduction in NrCAM protein levels occurs through cell-autonomous or non-cell-autonomous mechanisms. In addition, it is unknown whether NrCAM protein levels are also dysregulated in *Fmr1* knockout neurons. Future studies should investigate whether the reduction in astrocytic NrCAM levels in *Fmr1* knockout mice is associated with impairments in inhibitory synapse formation and function.

**Table 1:** Overview of changes in ICAM5, DSCAM, NCAM2, Thy-1 and NrCAM expression in Fragile X syndrome models. The mRNA and protein levels are described as either upregulated (↑) or downregulated (↓).

IgSF CAM	Experimental Model	mRNA Levels	Protein Levels	Study Reference
ICAM5	Hippocampus, prefrontal cortex and amygdala of 3-week-old *Fmr1* knockout mice.	No change.	↑	[175]
DSCAM	*Fmr1^null^ Drosophila* larval and adult brains.	No change.	↑	[176]
NCAM2	Isogenic *FMR1*^y/−^ hPSC-derived excitatory neurons.	Not measured.	↑	[191]
	Hippocampus of *Fmr1* knockout mice (in response to early environmental enrichment).	↑	Not measured.	[194]
Thy-1	Detergent-resistant membranes from the brain tissue of adult *Fmr1* knockout mice.	Not measured.	↓	[196]
NrCAM	Cortical astrocytes from *Fmr1* knockout mice.	Not measured.	↓	[234]

## 4. Rett Syndrome and IgSF CAMs

Rett syndrome is a severe and progressive neurodevelopmental disorder that is one of the leading genetic causes of ID in females [238]. Patients with Rett syndrome appear to undergo normal development until 6 to 18 months of age, followed by the loss of purposeful hand skills and spoken language abilities [239,240,241]. In addition, Rett syndrome patients exhibit hand stereotypies, including hand wringing, mouthing and clapping [239,242,243]. Other characteristic features of Rett syndrome include microcephaly, breathing disturbances, ataxia, gait apraxia and seizures [238,244,245]. The global prevalence of Rett syndrome has been estimated to be 7.1 per 100,000 females [246]. To date, trofinetide, a synthetic analog of the N-terminal tripeptide (glycine–proline–glutamate) of insulin-like growth factor 1 (IGF1), was approved as the only therapy for Rett syndrome [247,248].

Over 95% of Rett syndrome cases are caused by loss-of-function variants in the *MECP2* gene, which is located on the X chromosome (Xq28), and encodes methyl-CpG binding protein 2 (MeCP2) [238,249]. While Rett syndrome primarily affects females, males with Rett syndrome caused by *MECP2* variants have also been reported [250,251,252,253]. Male patients exhibit more severe phenotypes than female patients, and generally die at a young age due to encephalopathy.

In gene promoters, the methylation of cytosine residues in CpG islands results in chromatin compaction, leading to transcriptional inactivation [254]. Studies have shown that MeCP2 is a transcriptional regulator, acting both as a repressor and activator. MeCP2 functions as a transcriptional repressor through binding to methylated DNA and recruiting co-repressor complexes, as reviewed in [255]. One co-repressor complex is composed of mSin3A and histone deacetylases [256,257]. The deacetylation of histone lysine residues by histone deacetylases promotes the condensation of chromatin, which represses transcription through inhibiting the binding of RNA polymerases [258]. In addition, MeCP2 interacts with the NCoR co-repressor complex to decrease the rate of transcriptional initiation in the brain [259].

However, MeCP2 also acts as a transcriptional activator. MeCP2 directly interacts with CREB1 and promotes the transcription of the somatostatin (*Sst*) gene [260]. MeCP2 also physically interacts with RNA polymerase II at transcription start sites, and acts as a positive cofactor [261]. In addition, MeCP2 directly interacts with the super elongation complex to facilitate the transcription of a subset of genes regulating synaptic plasticity in the mouse brain [262]. Furthermore, MeCP2 is also involved in the regulation of chromatin architecture, mRNA alternative splicing and miRNA processing [263,264,265,266].

MeCP2 is expressed in numerous tissues, including the brain, heart, lungs, kidneys, liver, spleen and skeletal muscle [267,268]. In the brain, MeCP2 is widely expressed, and is present in neurons, astrocytes, microglia and oligodendrocytes [267,268,269,270,271,272,273]. Strikingly, in neuronal nuclei, the abundance of MeCP2 is close to that of the histone octamer [274]. MeCP2 is essential for proper neuronal development and maturation. In neurons, MeCP2 is involved in dendritic growth and arborization [74,275,276,277], dendritic spine formation [74], and excitatory synapse formation [278,279,280,281]. Importantly, the expression of MeCP2 in glial cells, including astrocytes and microglia, is required for correct neuronal development and function [269,271,282]. Indeed, glial cells are recognized to contribute to the pathogenesis of Rett syndrome through causing neuronal dysfunction in a non-cell autonomous manner, as reviewed in [283].

Neuropathological studies using post-mortem brain tissue samples from Rett syndrome patients found that neurons exhibit reduced dendritic complexity and dendritic spine density [74,75,284]. These deficits have also been observed in neurons from mouse models of Rett syndrome [83,89,285,286,287]. In addition, forebrain neurons differentiated from *MECP2* mutant hESC and iPSC lines display reduced neurite outgrowth and dendritic complexity, and impaired mitochondrial function [288]. Furthermore, iPSC-derived neurons from Rett syndrome patients have decreased densities of excitatory synapses and lower frequencies of spontaneous postsynaptic currents [87]. Importantly, trofinetide was designed to improve synaptic function in MeCP2-deficient neurons [89,247,289].

A growing body of evidence indicates that MeCP2 regulates the expression of CAMs that are important for neuronal development and synaptic function. In this review, we discuss the links between MeCP2 and IgSF CAMs L1CAM, KIRREL2, KIRREL3, contactin 4 (CNTN4) and NEGR1, and their implications in Rett syndrome.

### 4.1. L1CAM in Rett Syndrome

L1CAM is encoded by the *L1CAM* gene located on chromosome X (Xq28) [290]. During nervous system development, L1CAM promotes neurite outgrowth [291,292,293,294]. MeCP2 directly interacts with and regulates the expression of L1CAM [295,296]. L1CAM protein levels are reduced in neural precursor cells (NPCs) derived from a male Rett syndrome patient carrying a nonsense mutation in the *MECP2* gene (Table 2) [295]. In addition, neurite outgrowth and cell survival are reduced in NPCs derived from this patient. The deficits in neuritogenesis in the Rett syndrome NPCs are rescued by the overexpression of MeCP2 or L1CAM.

The human *L1CAM* gene has two promoters, with both promoters containing a CpG island [297]. Additionally, the *L1CAM* promoter contains E-box domains that act as binding sites for the Slug/Snail transcription factors [298]. It was shown using the luciferase reporter assay that MeCP2 binds to a single E-box domain in the *L1CAM* promoter and enhances transcription [295]. These findings highlight a functional relationship between MeCP2 and L1CAM in promoting neurite outgrowth in NPCs.

The proteolytic cleavage of L1CAM generates a fragment consisting of the intracellular domain [299]. The L1CAM intracellular fragment is imported into the nucleus, where it interacts with numerous nuclear proteins, including MeCP2, histone H1.4 and topoisomerase I. The intracellular domain of L1CAM binds directly to MeCP2 through the sequence motif KDET [296]. Proximity ligation assays demonstrated that MeCP2 closely associates with L1CAM in the nucleus and cytoplasm of cultured cortical and cerebellar neurons. MeCP2 cooperates with L1CAM to promote neurite outgrowth and cell migration. The siRNA knockdown of MeCP2 expression strongly reduces L1CAM-dependent neurite outgrowth in cultured cortical neurons. In addition, MeCP2 is required for the L1CAM-mediated migration of HEK293 cells.

Furthermore, the siRNA-induced knockdown of MeCP2 or L1CAM expression in cortical neurons leads to reduced mRNA and protein levels of myocyte enhancer factor 2C (Mef2c), a transcription factor belonging to the MCM1, agamous, deficiens, and serum response factor (MADS) box family [296,300]. Mef2c plays various roles in the early development of neural cells [300]. In nestin-expressing neural progenitor cells, the conditional knockout of Mef2c in vivo impairs neuronal differentiation [301]. It would be valuable to investigate whether MeCP2 and L1CAM cooperate to regulate the expression of other genes known to play important roles in neuronal development.

### 4.2. KIRREL2, KIRREL3 and CNTN4 in Rett Syndrome

In the mouse olfactory system, olfactory sensory neurons expressing the same odorant receptor project their axons onto a few topographically fixed glomeruli in the olfactory bulb [302,303]. The correct convergence of olfactory sensory axons to their target glomeruli relies on the activity-dependent expression of IgSF CAMs including KIRREL2 (containing 5 Ig-like domains in its extracellular domain (Figure 1)), KIRREL3 (containing 5 Ig-like domains in its extracellular domain (Figure 1)) and CNTN4 (containing 6 Ig-like and 4 FNIII domains in its extracellular domain (Figure 1)) [302,304,305,306].

Interestingly, a study found that the mRNA transcript and protein levels of KIRREL2, KIRREL3 and CNTN4 were increased in MeCP2-deficient olfactory sensory neurons (Table 2) [307]. Through ChIP-seq analysis, it was shown that MeCP2 binds to the *KIRREL2* gene promoter CpG island, along with exons and introns in the gene. In contrast, minimal MeCP2 binding was observed for the *KIRREL3* and *CNTN4* genes. The authors demonstrated that MeCP2 is an important regulator of activity-dependent KIRREL2 expression. In response to odor-evoked neuronal activity, the binding of MeCP2 to the *KIRREL2* gene (including the promoter CpG island) was strongly reduced. This reduction was associated with an upregulation of *KIRREL2* mRNA levels in olfactory sensory neurons.

The findings of this study indicate that MeCP2 represses *KIRREL2* transcription in the basal state, and that this transcriptional repression is alleviated during neuronal activity. However, additional research is needed to uncover the molecular mechanisms that increase the expression of KIRREL3 and CNTN4 in MeCP2-deficient olfactory sensory neurons.

### 4.3. NEGR1 in Rett Syndrome

miRNAs regulate multiple aspects of brain development, including neurogenesis, axonal outgrowth and synaptogenesis [308]. The dysregulation of miRNA expression has been linked to neurodevelopmental disorders, including Rett syndrome [308,309,310,311]. Studies have shown that MeCP2 represses the transcription of various miRNAs [311,312,313]. In addition, MeCP2 regulates the nuclear processing of miRNAs by interacting with the Drosha microprocessor complex [265,266].

It was reported that miR-203 was upregulated in the prefrontal cortex of MeCP2-deficient mice compared to control littermates [310]. Reduced neurite outgrowth and dendritic arborization are features of Rett syndrome [288,314]. Intriguingly, an earlier study showed that miR-203 directly inhibits the translation of NEGR1, an IgSF member containing 3 Ig-like domains (Figure 1), through binding to the 3′ UTR region of the *NEGR1* gene, leading to suppressed neurite outgrowth [315]. In mouse primary cortical neurons, inhibiting miR-203 leads to an upregulation in NEGR1 mRNA and protein levels, along with an increase in neurite outgrowth.

To date, there are no studies demonstrating that NEGR1 expression is dysregulated in MeCP2-deficient mice or other Rett syndrome models. In addition, there is no evidence showing that NEGR1 can rescue deficits in neurite outgrowth in Rett syndrome. Further, it is unknown whether MeCP2 directly regulates the transcription of the *NEGR1* gene. Therefore, we propose a mechanism where MeCP2 indirectly regulates the expression of NEGR1 through miR-203. However, this mechanism needs to be experimentally validated. Future research should focus on the functional role of NEGR1 in the pathogenesis of Rett syndrome, and whether alterations in *NEGR1* gene expression contribute to the impairments in neurite outgrowth observed in Rett syndrome patients.

**Table 2:** Overview of changes in L1CAM, KIRREL2, KIRREL3 and CNTN4 expression in Rett syndrome models. The mRNA and protein levels are described as either upregulated (↑) or downregulated (↓).

IgSF CAM	Experimental Model	mRNA Levels	Protein Levels	Study Reference
L1CAM	Neural precursor cells derived from a male Rett syndrome patient carrying a nonsense mutation in the *MECP2* gene (Q83X).	Not measured.	↓	[295]
KIRREL2	Olfactory sensory neurons from *Mecp2*^−/y^ mice.	↑	↑	[307]
KIRREL3	Olfactory sensory neurons from *Mecp2*^−/y^ mice.	↑	↑	[307]
CNTN4	Olfactory sensory neurons from *Mecp2*^−/y^ mice.	↑	↑	[307]

## 5. Down Syndrome and IgSF CAMs

Down syndrome (DS) or trisomy 21 is the most prevalent genetic cause of ID, and results from the triplication of all or part of human chromosome 21 (HSA21) [55,316,317,318]. As the most commonly occurring aneuploidy in humans [318], DS affects approximately 1 in 700 to 1 in 800 live births globally [319], and is typically diagnosed prenatally or at birth [320]. The clinical features of DS include short stature, muscle hypotonia, distinctive craniofacial features, congenital heart defects and ID [55,321]. In addition, individuals with DS are susceptible to developing other health conditions including epilepsy, leukemia, autoimmune diseases, hearing and vision problems, and early-onset Alzheimer’s disease [55]. Importantly, the lifetime risk of dementia in DS patients is over 90%, with adults with DS exhibiting the neuropathological features of Alzheimer’s disease, including amyloid-β (Aβ) plaques and neurofibrillary tangles, by the age of 40 years [322].

DS results in a mild to moderate ID that is linked to abnormalities in synaptic development observed in fetuses [323] and children [324,325]. These abnormalities are not compensated until adulthood, with dendritic atrophy reported to occur in adults with the condition [326]. DS is accompanied by a marked decrease in the number of dendritic spines [327,328]. At the ultrastructural level, the pre- and post-synaptic width of synapses is reduced in DS patients [327]. At the molecular level, impaired synaptogenesis in DS is accompanied by decreased levels of synaptic markers, such as synaptosomal-associated protein 25 [329] and drebrin [330,331]. Dendritic spine anomalies have also been observed in mouse models of DS. Cultured cortical neurons from Ts65Dn and Ts2Cje mice have decreased densities of dendritic spines, and also reduced numbers of mature, mushroom-shaped spines [332]. Further, iPSC-derived neurons from a DS patient failed to undergo LTP and LTD [333].

To date, there is no cure for DS [334], nor are there any approved pharmacological therapies for treating the life-long ID in DS [335]. The current interventions for DS are symptom-oriented, including physiotherapy, occupational and speech therapy and specialized education programs [334]. HSA21 contains 235 protein-coding genes [336]. Several genes encoding IgSF CAMs are located on HSA21 including *DSCAM* (region 21q22.2–22.3) and *NCAM2* (region 21q21.1), which are both highly expressed in the brain [103,337,338].

Studies have implicated both DSCAM and NCAM2 in the pathophysiology of DS. Advancing our knowledge as to how dosage imbalances of the *DSCAM* and *NCAM2* genes contribute to DS phenotypes can assist researchers in identifying relevant molecular pathways and the development of therapeutic interventions.

### 5.1. NCAM2 in DS

Multiple studies have shown that *NCAM2* gene expression is increased in the DS brain. An early study using whole-genome microarray analysis found that *NCAM2* gene expression was upregulated in the dorsolateral prefrontal cortex of adult DS patients compared to healthy individuals [339]. In addition, Palmer and colleagues performed single-nucleus transcriptome analysis of the postnatal human DS brain [340]. They analyzed cerebral cortical Brodmann Areas 8 and 9 from DS and control brains using single-nucleus RNA sequencing (snRNA-seq). Notably, in DS patients, *NCAM2* gene expression was upregulated in excitatory neurons, inhibitory neurons, astrocytes, oligodendrocytes and oligodendrocyte precursor cells.

Furthermore, a proteomics analysis found that the levels of NCAM2 were increased in both brain tissue and cerebrospinal fluid of DS patients compared to healthy individuals [341]. The correct gene dosage of *NCAM2* is critical for proper excitatory synapse development [192]. Consequently, the deficits in synaptogenesis observed in the DS brain may be linked to dosage imbalances of the *NCAM2* gene. It would be valuable to investigate whether reducing NCAM2 expression through genetic or pharmacological means ameliorates the synaptic and cognitive deficits in DS patients.

#### NCAM2 in Early-Onset Alzheimer’s Disease

Individuals with DS are at a great risk of developing early-onset Alzheimer’s disease, with adults with DS having Aβ plaques in the brain by 40 years of age [55,322]. The Aβ peptide is generated by the intramolecular cleavage of the amyloid precursor protein (APP) by β-site amyloid precursor protein cleaving enzyme 1 (BACE1) [342]. Like the *NCAM2* gene, the *APP* gene is located on HSA21 (21q21.2-3) [343,344]. The increased dosage of the *APP* gene results in the upregulated expression of APP in the brains of DS patients [344,345].

It was recently shown that APP directly binds to the extracellular domain of NCAM2 [342]. Importantly, NCAM2 promotes the targeting of APP and BACE1 to Rab11-positive recycling endosomes in neurons, resulting in the amyloidogenic processing of APP and Aβ production [342]. The increase in NCAM2 levels in the brains of DS patients [339,340,341], may thus elevate the production of Aβ peptides and be a key factor contributing to the early-onset Alzheimer’s disease risk faced by people with DS. Together, these findings highlight a potential role for NCAM2 as a therapeutic target for treating Alzheimer’s disease in DS patients.

### 5.2. DSCAM in DS

DSCAM is an IgSF member containing 10 Ig-like and 6 FNIII domains in its extracellular domain (Figure 1). It is overexpressed in young individuals with DS [346] and studies in animal models and human neurons indicate that DSCAM overexpression can be one of the underlying causes of ID in this syndrome. Overexpression of DSCAM was linked to changes in neuronal growth, guidance and self-avoidance. In the *Drosophila* olfactory system, overexpression of Dscam in projection neurons causes dendrites to be more diffuse during development and shifts their relative position in adulthood [347]. In *Drosophila* longitudinal axons, which do not normally cross the midline, overexpression of Dscam induces ectopic midline crossing [348].

In the *Xenopus* visual system, overexpression of Dscam reduces dendritic branching and growth rate [349]. In mice, Dscam guides dendrite targeting in type 2 dopaminergic amacrine cells by restricting the stratum in which exploring retinal dendrites stabilize in a *Dscam* dosage-dependent manner, while Dscam overexpression in outer retinal cells is sufficient to retarget their neurites [350]. Dscam overexpression impairs development and axon guidance in mouse hippocampal pyramidal neurons and leads to impaired hippocampal-dependent learning and reduced anxiety in adult mice [351]. Normalization of DSCAM in DS human iPSC-derived neurons rescues deficits in axonal growth and guidance [351].

DSCAM overexpression also influences neuronal development outside of the brain. Variants in *DSCAM* show association with Hirschsprung disease, in which nerves are missing from parts of the intestine, and the incidence of this disease is higher in children with DS [352]. In *Drosophila*, Dscam is expressed in the enteric/stomatogastric nervous system and its overexpression results in frontal and hindgut nerve overgrowth [353]. Dscam is also expressed in the Zebrafish gut. Knockdown of Dscam causes a reduction in number of enteric neurons in the hindgut of zebrafish; however, overexpression of DSCAM had no effects on neuron number in this system [352].

In addition to effects on neuronal development, overexpression of DSCAM was linked to changes in synapse numbers and function. Elevated Dscam protein levels in flies with three copies of the *Dscam* gene produce synaptic targeting errors in a hard-wired neural circuit, which impair the flies’ sensory perception [176]. Overexpression of DSCAM in the *Drosophila* larval class IV dendritic arborization (C4da) neurons leads to increased growth of presynaptic terminals [177], which is caused by increased activity of Abl kinase [187]. In *Drosophila* larvae, expression of three copies of *Dscam* changes the properties of spontaneous and electrically evoked transmitter release at the glutamatergic neuromuscular junction, strengthens short-term synaptic depression during high-frequency firing of the motor nerve, and impairs locomotor coordination despite preserved gross motor function [354].

DSCAM is overexpressed in the Ts65Dn mouse model for DS. In these mice, GABAergic innervation of neocortical pyramidal neurons by basket and chandelier interneurons is increased. Genetic normalization of DSCAM expression rescues the excessive GABAergic innervation and the increased inhibition of neocortical pyramidal neurons. Conversely, loss of DSCAM impairs GABAergic synapse development and function [355]. Interestingly, DSCAM is overexpressed in brains of APP transgenic mice, suggesting that DSCAM may also be involved in the early development of Alzheimer’s disease in DS patients [356,357]. Indeed, overexpression of DSCAM in mice leads to increased cell death in the retina [350].

## 6. Autism Spectrum Disorder and IgSF CAMs

Autism spectrum disorder (ASD) is a neurodevelopmental disorder characterized by persistent impairments in social communication and interaction along with repetitive, restricted behavior patterns and interests [32]. Symptoms for ASD manifest during early development, usually between 12–24 months of age, but can also appear earlier (from 6 months of age) if they are more severe [32,358]. To date, there are no established diagnostic biomarkers for ASD, and the clinical diagnosis of ASD is based on behavioral phenotypes [359,360]. The diagnosis of ASD by clinicians is currently based on developmental history, physical examinations, direct observation of behavior in different settings, and the use of standardized assessment tools including checklists/questionnaires and structured diagnostic interviews [359,361].

ASD is estimated to affect 0.5–1% of the global population, with prevalence estimates increasing over time [362,363]. ASD often co-occurs with ID, with 33% of individuals with ASD also having ID [40,363,364]. In addition, ASD frequently co-occurs with other neuropsychiatric conditions, including schizophrenia, major depressive disorder and epilepsy [365,366,367]. Currently, there are no approved pharmacological treatments for treating the core symptoms of ASD [361,368]. The etiology of ASD is multifactorial, with both environmental and genetic factors playing a role [369,370]. Numerous environmental factors including advanced parental age, bacterial and viral infection during pregnancy, medication use, maternal metabolic conditions, premature birth and exposure to air pollutants are associated with an increased risk of ASD [371].

Furthermore, genetic factors play an important role in the pathogenesis of ASD [372]. ASD is one of the most heritable neurodevelopmental disorders [373]. Twin studies have shown that ASD is highly familial, with monozygotic twins more likely to share an ASD diagnosis compared to dizygotic twins [373,374]. A 2009 study found that the concordance rate for ASD was 88% in monozygotic twins and 31% in dizygotic twins [375]. A later study found that the concordance rate for ASD was 95.2% in monozygotic twins and 4.3% in dizygotic twins [376].

ASD can be idiopathic or syndromic [369,377]. Idiopathic ASD refers to autism which does not have an identifiable cause and represents the majority (about 85%) of ASD cases [377,378]. In contrast, syndromic ASD refers to autism cases with a known genetic cause, including chromosomal aberrations or monogenic variants [369]. Examples of syndromic forms of ASD include Down syndrome, Fragile X syndrome, Rett syndrome and tuberous sclerosis complex [369,379,380]. ASD is extremely genetically heterogeneous, and can be caused by both inherited and de novo gene variants [374,381]. While ASD can be attributed to monogenic causes, the majority of ASD patients have multiple genes that contribute to the pathogenesis of this disorder [360].

Numerous large-scale genomic studies, including genome-wide association studies, whole-exome sequencing and whole-genome sequencing, have been conducted on ASD patients and their families, with more than 100 genes being linked to ASD [374,380,382,383,384]. Different types of genetic variants have been implicated in ASD pathology including chromosomal abnormalities, CNVs and SNVs [374,380]. Despite the immense genetic heterogeneity of ASD, many ASD risk genes converge onto specific biological processes including synapse formation and function, chromatin remodeling and transcriptional regulation [68,380,385,386,387,388].

Impairments in synapse formation, pruning and plasticity have been linked to the pathology of ASD [389,390,391]. Consequently, a feature of ASD is alterations in neural connectivity, which is thought to underlie the behavioral symptoms observed in this disorder [389,390,392,393]. Notably, many of the ASD candidate genes encode for CAMs [24,99,394,395,396,397,398,399,400,401,402,403], including IgSF members such as contactins, the L1CAM family, SynCAMs, NCAMs and KIRREL3 [24,99,397,398,404,405,406,407,408,409,410], which are discussed below.

### 6.1. NrCAM in ASD

NrCAM is encoded by the *NRCAM* gene which lies within the chromosome 7q31.1 autism-susceptibility region AUTS1. Several genetic association studies have linked *NRCAM* SNPs to ASD (Table 3) [411,412,413]. A specific *NRCAM* haplotype was associated with severe obsessive-compulsive behaviors in a subset of autism families [412]. *NRCAM* was also identified among genes showing predicted ataxin 2-binding protein 1 (A2BP1)-dependent differential splicing in ASD brain tissue, suggesting that altered *NRCAM* isoform regulation may contribute to ASD-related molecular changes [414]. Within an L1CAM-related interaction network, NrCAM was identified as a first-degree interactor of neurofascin [415]. A recurrent rare variant of neurofascin was identified in ASD families and proposed to contribute to ASD risk alongside variants in interacting genes [415].

Studies of NrCAM-null mice further support a role for NrCAM in ASD-related behaviors (Table 3). Loss of NrCAM leads to reduced sociability, impaired reversal learning and reduced prepulse inhibition, with some effects differing between male and female mice [236]. NrCAM-null mice also show changes in impulsivity, reaction to stress and passive-avoidance learning [416]. NrCAM-deficient mice exhibit brain malformations, such as a mild reduction in the size of cerebellar lobes [417]. Mice deficient for both L1CAM and NrCAM exhibit more severe cerebellar defects, including cerebellar dysgenesis and reduced thickness of the inner granule cell layer [417].

### 6.2. CHL1 in ASD

CNVs or missense polymorphisms in the *CHL1* gene located at chromosome 3p26.3 are risk factors for ASD, ID, schizophrenia and epilepsy (Table 3) [418,419,420]. The CHL1 protein is a member of the IgSF and L1 family and contains 6 Ig-like and 5 FNIII domains in its extracellular domain. Cases involving duplications or deletions of *CHL1* show cognitive and language impairment, learning difficulties, microcephaly, motor delay and behavioral abnormalities [419,421,422,423,424].

A rare 3p26.3 microduplication located upstream of *CHL1* was inherited by several families with ASD, suggesting that altered regulation of *CHL1* may contribute to ASD susceptibility [425]. A *CHL1* microduplication was also reported in an individual with ASD and developmental delay [426]. A deletion involving both *CHL1* and contactin 6 (*CNTN6*) was reported in ASD families, suggesting that cell adhesion pathways may contribute to ASD risk [427]. Chromosomal microarray studies have also detected duplications and deletions involving *CHL1* in individuals with ASD, developmental delay, seizures and language impairment [428,429,430].

CHL1-deficient mice show several behavioral abnormalities related to ASD and other neuropsychiatric disorders (Table 3). CHL1 regulates dopamine D2-receptor-dependent functions in mice, and CHL1 deficiency alters locomotor activity and behavioral responses to D2-receptor drugs [431]. Chronic mild stress disrupts latent inhibition and neuronal activation in CHL1-deficient mice, modeling schizophrenia-like learning and attention impairments [432]. CHL1 also supports maintenance of inhibitory circuits and synaptic plasticity in the mouse hippocampus [433]. CHL1-deficient mice show an age-dependent reduction in hippocampal parvalbumin-expressing interneurons, which may disrupt cognition and the balance between excitatory and inhibitory signaling [433].

Many ASD-associated CNVs are heterozygous and can alter gene dosage [434]. The association of both *CHL1* duplications and deletions with neurodevelopmental abnormalities suggests that *CHL1* may be dosage-sensitive. However, *CHL1* CNVs inherited from unaffected parents demonstrate incomplete penetrance [421,422,423,426], complicating genetic counseling. The potential mechanistic relationship between L1CAM family members and ASD remains to be established.

### 6.3. NCAM2 in ASD

Multiple studies have associated NCAM2 with ASD (Table 3) [407,409,410,435,436]. A genetic linkage analysis of autism-affected relative pairs with a history of developmental regression identified a susceptibility locus located on the long arm of chromosome 21 (21q), and proposed *NCAM2* as a candidate gene due to its functions in the nervous system [407]. A genome-wide association study also proposed *NCAM2* as an ASD candidate gene [409]. In addition, Gai and colleagues performed a modified case–control CNV study focusing on families of European descent with a child diagnosed with idiopathic autism [435]. They identified an inherited hemizygous intronic deletion in the *NCAM2* gene in autism patients that was not present in healthy control individuals.

It has also been reported that an ASD patient carried a microdeletion containing the entire *NCAM2* gene and no other functional genes [410]. Another report found that a male with ASD carried a 21q21 deletion consisting of 19 genes including *NCAM2* along with a complex chromosomal rearrangement [436]. As the patient had chromosomal translocations, it is possible that these could have contributed to his phenotype in addition to the *NCAM2* gene deletion. Further, NCAM2-deficient mice have impairments in short-term memory and cognitive flexibility, along with autistic behaviors (Table 3) [437].

Deletions of the *NCAM2* gene have also been found in patients with ID. There are a small number of global developmental delay/ID patients with heterozygous deletions involving the entire *NCAM2* gene [438,439]. The *NCAM2* gene is highly likely to be intolerant to loss-of-function mutations, having a probability of loss-of-function intolerance (pLI) score of 1 [440,441]. Genes with a pLI ≥ 0.9 are commonly linked with haploinsufficiency [441,442]. NCAM2 deficiency impairs neuronal development both in vitro and in the mouse brain [437,443]. Knockdown of NCAM2 expression in cultured hippocampal neurons leads to abnormalities in both dendritic and axonal development [443]. Further, silencing NCAM2 expression in the mouse cortex leads to impaired neuronal migration and dendritic tree formation. Lastly, NCAM2-deficient mice have axon guidance defects in the hippocampus [437].

These studies establish that the appropriate levels of the NCAM2 protein are essential for proper brain development. However, further research is required to uncover the mechanisms through which CNVs of the *NCAM2* gene contribute to the development of ASD.

### 6.4. DSCAM in ASD

*DSCAM* variants are associated with ASD (Table 3) [388,444,445], can reduce DSCAM expression or function [445,446], and are often accompanied by ID of variable severity [445]. Accordingly, the loss of DSCAM affects neuronal development in various model systems. In the *Drosophila* olfactory system, Dscam acts in projection neurons and local interneurons to control the elaboration of dendritic fields. The removal of Dscam selectively from projection neurons or local interneurons leads to clumped dendrites and a marked reduction in their dendritic field size [347]. In *Drosophila* dendrite arborization neurons, Dscam controls cell-intrinsic aspects of dendrite guidance. Loss of Dscam in single neurons causes a strong increase in self-crossing. Overexpression of the same Dscam isoforms in two neurons with overlapping dendritic fields forces a spatial segregation of the two fields, supporting the model that dendritic branches of neurons use isoform-specific homophilic interactions to ensure minimal overlap [447].

In the *Xenopus* visual system, downregulating DSCAM in tectal neurons significantly increases dendritic growth and branching rates while inducing dendrites to take on tortuous paths. However, DSCAM knockdown in retinal RGCs leads to a lower growth of axons and relatively simple morphology. Tectal DSCAM knockdown leads to irregular behavioral responses to visual stimuli in swimming tadpoles. These functional deficits correspond with changes in expression of markers of excitatory and inhibitory transmission VGLUT and VGAT in the tectum [349].

To regulate axonal and dendritic growth, DSCAM interacts with Netrin-1, a guidance cue. In *Drosophila*, simultaneous genetic knockout of the two *Dscam* genes and the Netrin receptor fra produces a midline crossing defect [348]. In chicks, the knockdown of DSCAM inhibits netrin-induced axon outgrowth and commissural axon turning in vitro and causes defects in commissural axon projection and pathfinding in vivo [448]. DSCAM associates with uncoordinated-5C (UNC5C), and the knockdown of DSCAM or UNC5C by specific shRNAs or blocking their signaling by overexpressing dominant negative mutants suppresses netrin-1-induced growth cone collapse in mice [449].

DSCAM is also involved in regulating synapse formation. In *Drosophila* larval C4da neurons, the loss of Dscam leads to reduced growth of presynaptic terminals [177]. The extracellular domain of DSCAM interacts with neuroligin 1 and blocks the neuroligin 1–neurexin1β interaction, which promotes synapse formation. A complete loss of DSCAM expression in DSCAM-deficient mice leads to premature spine maturation and autism-like behaviors [450]. The loss of one copy of *DSCAM* in mice also leads to hyperactivity, increased anxiety-like behavior, motor coordination deficits, and impairments in hippocampal-dependent learning and memory, including impairments in working memory, long-term memory, and contextual fear learning (Table 3) [451].

Neural stem cell-specific *Dscam* heterozygous knockout mice show deficits in social interaction and social memory and reduced NMDA receptor currents [446]. NMDA receptor-mediated currents were also significantly reduced in iPSC-derived telencephalic induced neuronal cells from an ASD patient with a heterozygous point mutation in the *DSCAM* gene, leading to a reduction in the mRNA and protein DSCAM levels in dendrites [446]. DSCAM knockdown in control induced neuronal cells also resulted in the downregulation of NMDA receptors [446].

**Table 3:** Summary of genetic and animal studies for NrCAM, CHL1, NCAM2 and DSCAM.

**Neuronal Cell Adhesion Molecule (*NRCAM*), 7q31.1**				
**Study Reference**	**Method/Number of Participants**	**Variants Identified**	**ID Exclusion** **Criteria**	**Animal Studies**
[411]	Transmission disequilibrium test (TDT); haplotype analysis. 219 families (affected sibling pairs).	SNPs: Promoter: rs1269622 (C/T), rs3763463 (C/T) Intron 2: rs722519 (A/G) Haplotypes including rs722519 and rs3763463.	Excluded individuals with a performance IQ < 35.	NrCAM-null mice show reduced sociability, acquisition of a spatial task, impaired reversal learning and reduced pre-pulse inhibition [236], along with altered impulsivity, reaction to stress and passive-avoidance learning [416]. NrCAM-deficient mice exhibit brain malformations, such as mild reduction in the size of cerebellar lobes [417].
[412]	TDT and pedigree disequilibrium test (PDT); haplotype analysis. 352 families.	Overtransmission of haplotype G-G-A-G-C-A for rs722519-rs1269622-rs405945-rs6958498-rs401433-rs439587.	There were no exclusion criteria for ASD patients that were comorbid for ID.	
[413]	Case–control study; TDT in trios; haplotype analysis. 170 individuals (case–control) and 148 trios of families (TDT).	SNPs (case–control): Intron 1: rs1859767 (A/T), rs1034825 (T/C), rs2300045 (T/C), rs2300043 (T/C) Intron 3: rs1269655 (G/C), rs1269642 (T/G) Exon 11: rs1269621 (A/G) Haplotypes: rs3763463, rs1859767, rs1034825, rs2300045, rs2300043: C-A-C-T-C, C-T-T-C-G rs3763463, rs1859767, rs1034825, rs2300045, rs2300043, rs2300039, rs722519, rs2216259: C-T-T-C-T-T-G-C rs1269655, rs1269642: G-T, C-G	There were no exclusion criteria for ASD patients that were comorbid for ID.	
**Close homolog of L1 (*CHL1*)*,* 3p26.3**				
**Study Reference**	**Method/Number of participants**	**Variants identified**	**ID exclusion** **criteria**	**Animal studies**
[425]	Genome-wide CNV analysis. 382 individuals from 40 extended families.	Duplication of 3p26.3 including *CHL1*.	Excluded individuals with an IQ < 35 or developmental level < 18 months.	CHL1-deficient mice show altered locomotor activity and behavioral responses to D2-receptor drugs [431], reduced novelty reaction and spatial perception [452], and an age-dependent reduction in hippocampal parvalbumin-expressing interneurons [433]. CHL1 also supports maintenance of inhibitory circuits and synaptic plasticity in the mouse hippocampus [433]. Conditionally CHL1-deficient mice show impaired working memory, despite normal selective attention [453].
[427]	Genome-wide CNV analysis. 1063 families/1301 individuals.	Deletion of 3p26.3 that deletes *CHL1* and *CNTN6*.	Excluded individuals with an IQ < 35 or developmental level < 18 months.	
**Neural cell adhesion molecule 2 (*NCAM2*), 21q21.1**				
**Study Reference**	**Method/Number of participants**	**Variants identified**	**ID exclusion** **criteria**	**Animal studies**
[407]	Linkage analysis. 34 autism-affected relative pairs with a history of developmental regression.	Evidence for linkage was found on the long arm of chromosome 21 (21q).	Families were excluded if there was a known history of Fragile X syndrome or another chromosomal abnormality.	NCAM2-deficient mice display autistic behaviors in a sex-dependent manner [437]. Male but not female NCAM2-deficient mice exhibit repetitive digging behavior and increased olfactory acuity. In addition, NCAM2-deficient mice have impairments in short-term memory and cognitive flexibility.
[409]	GWAS noise reduction (GWAS-NR). GWAS-NR was applied to a family-based autism GWAS of 597 families and a second autism GWAS of 696 families from the Autism Genetic Resource Exchange.	*NCAM2* was identified as an ASD candidate gene.	Individuals with an IQ < 35 or developmental level < 18 months were excluded.	
[435]	Modified case–control CNV study, restricting inclusion to probands and siblings of European descent with a diagnosis of idiopathic autism. The discovery cohort included 1793 subjects (631 cases and 1162 parental samples). The replication cohort included 1702 subjects (593 cases and 1109 parents).	An inherited hemizygous intronic deletion in the *NCAM2* gene was found in autism patients that was not present in healthy control individuals.	Individuals with known genetic syndromes or other non-idiopathic causes of autism were excluded.	
[410]	Array-based comparative genomic hybridization (array-CGH) analysis and multiplex ligation-dependent probe amplification (MLPA analysis). Genetic testing was performed on an ASD patient and 8 of his family members.	A heterozygous 1.6 Mb deletion on 21q21.1-q21.2 was detected in the ASD patient. This deleted region contained the entire *NCAM2* gene and two long intergenic non-protein coding RNAs (LINC00317 and LINC00308). The patient’s mother and maternal grandmother also carried the *NCAM2* deletion, but did not have ASD.	This family genetic study did not specify any exclusion criteria for ID.	
**Down syndrome cell adhesion molecule (*DSCAM*), 21q22.2–q22.3**				
**Study Reference**	**Method/Number of participants**	**Variants identified**	**ID exclusion** **criteria**	**Animal studies**
[454]	Large-scale exome sequencing. 21,219 family-based samples (6430 ASD cases, 2179 unaffected siblings, and both parents). 14,365 case–control samples (5556 ASD cases, 8809 controls).	Through transmission and de novo association (TADA) analysis, *DSCAM* was identified as an ASD risk gene. 4 de novo protein-truncating variants in the *DSCAM* gene were found in ASD probands.	ASD patients that were comorbid for ID were included in this study. A subject was considered to have ID if they met any of the below criteria: Full-scale IQ < 70. The subject was administered but could not complete an IQ test. The subject had a human phenotype ontology term or International Classification of Diseases code indicating intellectual disability or mental retardation.	*DSCAM* heterozygous mice have impaired working memory, long-term memory and contextual fear learning [451]. Additionally, *DSCAM* heterozygous mice exhibit increased anxiety-like behavior, displayed hyperactivity and motor coordination deficits. DSCAM-deficient mice display autistic behaviors, including increased circling and grooming time, impaired social novelty and increased anxiety-like behavior [450]. In pyramidal neurons, DSCAM deficiency leads to the overmaturation of dendritic spines.
[388]	Whole-exome sequencing. 2517 families from the Simons Simplex Collection were sequenced. 2508 affected children, 1911 unaffected siblings and the parents of each family were analyzed.	3 de novo variants in the *DSCAM* gene in male children with autism were found.	There were no exclusion criteria for ASD patients that were comorbid for ID. Multiple IQ measures were recorded, including verbal, non-verbal and full spectrum. Probands were divided based on non-verbal IQ.	
		**Insertion (T):** 21:41414330:A:AT (frameshift variant).		
		**Substitution (A->T):** 21:41457527:A:T (splice-site variant).		
		**Insertion (TA):** 21:41457640:T:TTA (frameshift variant).		
[455]	Single-molecule molecular inversion probes. 208 candidate NDD (ASD, ID, and DD) disease-risk genes were sequenced. 13,407 probands with a primary diagnosis of ASD, ID or DD were screened. 6342 cases with a primary diagnosis of ASD and 7065 cases with a diagnosis of ID/DD were analyzed.	De novo likely gene-disruptive (LGD) mutations in the *DSCAM* gene were found in probands. **Proband:** NM_001389.3:c.5652_5653delAAinsAAA **Proband:** NM_001389.3:c.4885C>T **Proband:** NM_001389.3:c.4020delCinsCTA **Autism:** NM_001271534.1:c.1067del	Probands with ID were included in this study.	
[385]	Whole-exome sequencing The study began with 3976 ASD subjects (2303 trios) and 6059 unrelated controls. The exomes of the DNA samples were captured and sequenced. After variant calling, quality control was performed. The data was cleaned to 3871 subjects with ASD. Analysis for SNVs, CNVs and indels was then performed. For trios, analysis for de novo and transmission/non-transmission variants was performed.	Through transmission and de novo association (TADA) analysis, *DSCAM* was identified as an ASD risk gene.	There were no exclusion criteria for ASD patients that were comorbid for ID.	
[456]	Whole-genome sequencing. 5205 samples from families with ASD were sequenced. 3100 samples were from simplex families (one child with ASD) and 2105 samples were from multiplex families (two or more siblings with ASD). 1745 samples were from probands, while 879 samples were from affected siblings.	A frameshift variant within exon 8 of the *DSCAM* gene was found in an ASD patient. NM_001389:exon8:c.1533_1537del:p.K511fs NM_001271534:exon8:c.1533_1537del:p.K511fs	ASD patients that were comorbid for ID were included in this study. A total of 1102 patients had IQ data available, and 216 (19.6%) of these patients had ID (full-scale IQ < 70).	

## 7. L1 Syndrome

L1CAM, containing 6 Ig-like and 5 FNIII domains in its extracellular domain (Figure 1), is encoded by the *L1CAM* gene which maps to the q28 region of the X chromosome and contains 29 exons [457]. Mutations in *L1CAM*, the founding member of the L1CAM family, cause a disorder separate from ASD, known as L1 syndrome. L1 syndrome is an X-linked neurodevelopmental disorder spectrum characterized by HSAS (hydrocephalus with aqueductal stenosis), MASA syndrome (mental retardation, aphasia, shuffling gait, adducted thumbs), corpus callosum agenesis and X-linked spastic paraplegia [458]. *L1CAM* gene mutations account for 5% of all congenital hydrocephalus cases and represent the most frequent genetic cause of the pathology, affecting 1 in 30,000 male births [458]. Phenotypic expressivity is highly variable, even within families, ranging from mild spasticity or MASA features to severe congenital hydrocephalus and prenatal death [459].

**Figure 2:**
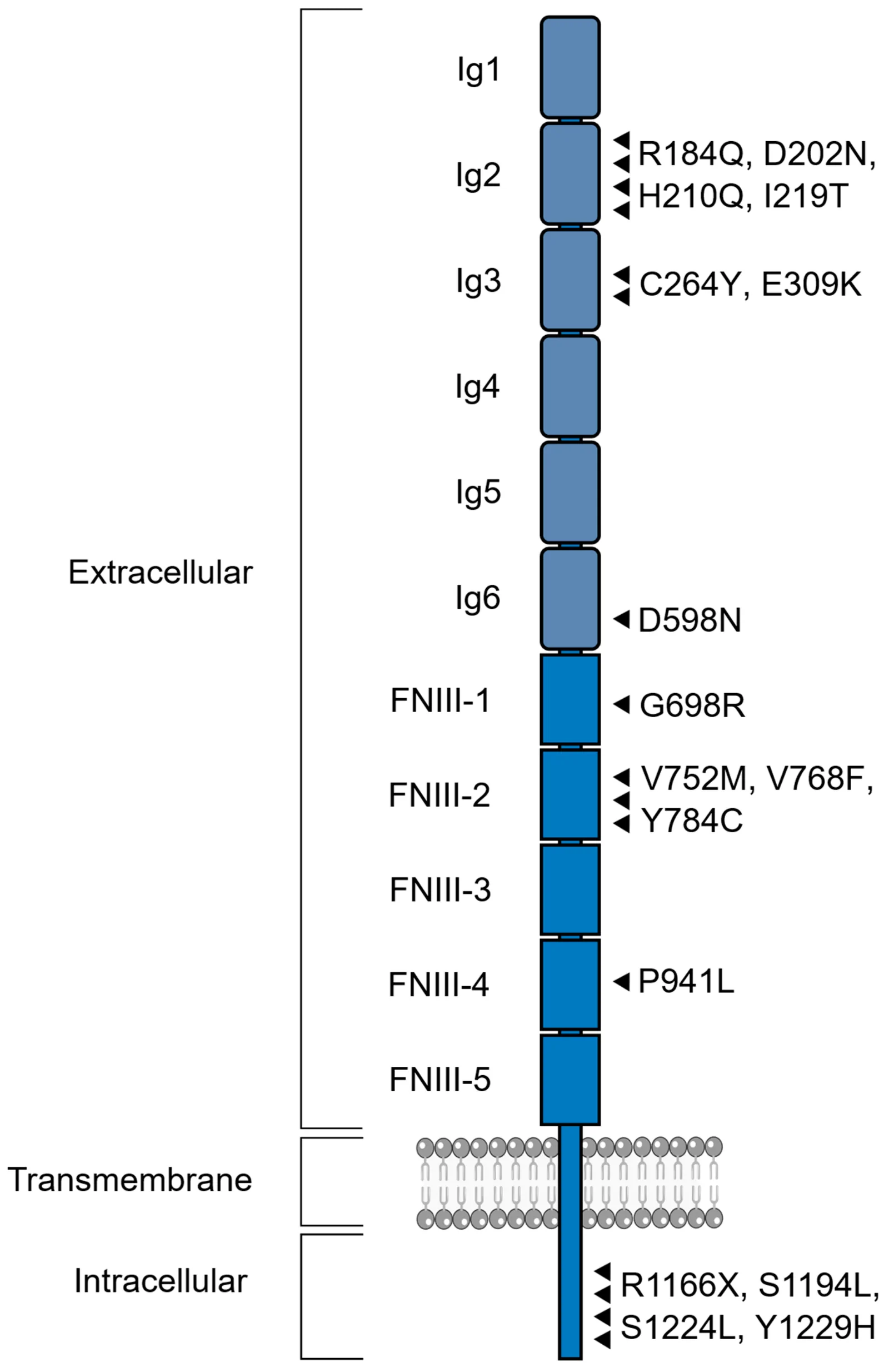
L1 syndrome-associated mutations. L1CAM contains six Ig-like motifs and five FNIII repeats in its extracellular domain, a transmembrane region and a cytoplasmic tail. Mutations L1-R184Q (VCV000009991.8), L1-D202N (VCV000689728.8), L1-H210Q (VCV000009988.1), L1-C264Y (VCV000009986.2), L1-E309K (VCV000838485.20), L1-D598N (VCV000009989.1), L1-G698R (VCV000265225.6), L1-V752M (VCV000009998.11), L1-Y784C (VCV000211340.6), L1-P941L (VCV001074836.9), L1-R1166X (VCV000647732.13), L1-S1194L (VCV000009993.9) and L1-S1224L (VCV001697985.7) were classified as pathogenic or likely pathogenic in ClinVar [466]. L1-I219T, L1-V768F and L1-Y1229H have been described as disease-associated mutations [470,471].

More than 300 *L1CAM* gene mutations have been reported, most commonly missense mutations, followed by small insertions or deletions, nonsense or frameshift mutations, and large deletions or duplications (Table 4) [460,461,462]. Nonsense or frameshift mutations that truncate extracellular domains or abolish L1CAM protein expression cause the most severe phenotypes [463]. Missense mutations that alter structurally important residues in the extracellular domain tend to cause more severe disease than mutations in the cytoplasmic domain [464,465]. Disease-causing *L1CAM* mutations are distributed across the protein, affecting multiple regions of its extracellular and intracellular domains (Figure 2) [466].

Extracellular domain mutations can disrupt L1CAM processing [467,468], cell surface expression [465,467,469,470], ligand binding [465,471] or signaling [472]. These effects vary by the affected domain and residue. Several Ig domain mutations predicted to destabilize the protein structure can cause intracellular retention and reduce cell surface expression [471]. The Ig2 mutant L1-R184Q is retained in the endoplasmic reticulum (ER), fails to sort effectively to ER exit sites and is restricted to the soma rather than the axon [467,469]. Similarly, the Ig6 mutant L1-D598N undergoes incomplete ER-to-Golgi maturation and impairs trafficking to the cell surface, reducing neurite outgrowth [469,473]. The Ig3 mutant L1-E309K also shows reduced surface expression [465,469,471,474] and impairs adhesion-dependent epidermal growth factor receptor (EGFR) activation [472], resulting in defective cell adhesion, migration and neurite outgrowth [473,474]. However, not all Ig domain mutants cause trafficking defects. Surface-exposed Ig2 mutants L1-H210Q and L1-I219T show moderate or increased surface expression relative to wildtype L1CAM [471,474], indicating they primarily affect extracellular interactions or signaling. Although Ig1-Ig4 domains form the primary horseshoe-shaped ligand-binding region [475], mutations in Ig5 and Ig6 can also alter the conformation of L1CAM and impair homophilic L1CAM binding or heterophilic binding with ligands such as TAX-1/axonin-1 [465,471]. L1-R184Q, L1-I219T, L1-H210Q and L1-D598N reduce homophilic binding [465,471]. While L1-R184Q and L1-I219T significantly impair TAX-1 binding, L1-D598N has a relatively modest effect and L1-H210Q increases TAX-1 binding [471]. L1-E309K maintains homophilic interactions but disrupts heterophilic TAX-1 binding [471]. FNIII domain mutations likewise produce variable effects on L1CAM functions. Mutations predicted to disrupt the domain structure, such as L1-G698R in FNIII-1, and FNIII-2 mutants L1-V752M, L1-V768F and L1-Y784C, cause intracellular retention and reduced surface expression [471]. The FNIII-2 mutants also disrupt homophilic L1CAM and heterophilic TAX-1 binding, indicating that the structural integrity of FNIII-2 is important for both ligand interactions and protein trafficking [471]. Mutations in FNIII-4 and FNIII-5 do not disrupt ligand binding but affect L1CAM processing [471]. Consistent with this, the membrane-proximal FNIII-4 mutant L1-P941L is retained in the ER and fails to mature through the Golgi [468]. This trafficking defect is associated with slower cell migration and diminished neurite outgrowth [471,474].

Intracellular domain mutations generally do not affect cell surface expression of L1CAM but disrupt cytoskeletal coupling, endocytosis, or signaling [469,470]. The cytoplasmic mutant L1-S1194L, located outside the SFIGQY ankyrin-binding motif, shows strong surface expression [469,470], undergoes normal post-translational modification, and supports neurite outgrowth [469]. In contrast, mutations within the ankyrin-binding motif, L1-S1224L and L1-Y1229H, reach the cell surface but reduce the ability of L1CAM to recruit ankyrin, disrupting L1CAM-cytoskeletal coupling [470]. L1-Y1229H also dysregulates L1CAM internalization [470]. The truncating nonsense mutant L1-R1166X lacks the cytoplasmic domain and consequently cannot recruit ankyrin or restore ankyrin levels at the axon initial segment [476]. Loss of ankyrin binding contributes to abnormal axonal tract development and synaptic organization [477]. A duplication mutant that alters the cytoplasmic domain of L1CAM also impairs signaling and trafficking, and has been associated with X-linked hydrocephalus [478].

Beyond point mutations, complete loss of L1CAM function has also been associated with L1 syndrome. A whole *L1CAM* gene deletion has been reported in an L1 syndrome patient [479]. Conditional deletion of *L1CAM* in human neurons impairs axonal elongation, dendritic arborization and action potential generation while reducing the levels of ankyrin G and ankyrin B [476]. AnkyrinB-deficient mice that exhibit L1 syndrome features, including hypoplasia of the corpus callosum and dilated ventricles, show reduced levels of L1CAM in axons [480].

*L1CAM*-null mice and mutation knock-in mice exhibit disease phenotypes similar to those observed in humans, such as hypoplasia of the corticospinal tract and cerebellar vermis [481,482], reduced corpus callosum size [482,483], ventricular dilation [481,482] and defects in axon guidance [484]. Transgenic mice carrying the Ig3 L1-C264Y mutation are smaller, have increased mortality, and show hypoplasia of the corticospinal tract, axon path-finding errors and impaired Schwann-cell–axon interactions, resembling L1-deficient mice [485]. L1-C264Y disrupts L1CAM trafficking and is incompletely processed, resulting in intracellular retention and reduced cell surface expression [485]. In contrast, mice carrying the Ig2 L1-D201N mutation, corresponding to the human L1-D202N mutation, maintain normal surface delivery but still develop L1 syndrome features, including increased neurological deficit scores and locomotion, as well as reduced neuronal migration and survival [483].

Mice carrying mutations in the first and third FNIII domains also exhibit brain malformations and behavioral changes [486,487]. FNIII-1 mutant mice show enhanced locomotion, altered social behavior, impaired motor coordination and hippocampal abnormalities [486]. FNIII-3 mutant mice exhibit reduced numbers of principal hippocampal neurons, increased neuronal death and astrogliosis, leading to obsessive-like behavior in male mice and reduced anxiety in female mice [487]. Together, different *L1CAM* mutations produce overlapping phenotypes related to L1 syndrome through distinct mechanisms.

**Table 4:** Mutations in L1CAM discussed in this review.

Mutation in L1CAM	Effect on Cell Surface Delivery	Effect on Homophilic Adhesion	Effect on Heterophilic Adhesion	Effect on Neurite Outgrowth
R184Q	↓ [471]	↓ [471,488]	↓ [465,471]	↓ [469,488]
D202N	~WT [483]	Not tested	Not tested	~WT [483]
H210Q	↑ in CHO [471] ↓ in PC12 [474]	↓ [465,471]	↑ [465,471]	↓ [488] ~WT [474]
I219T	↑ in CHO [471] ↓ in PC12 [474]	↓ [471]	↓ [471]	~WT [473,474]
C264Y	↓ [465,471,473,474]	↓ [465,471]	↓ [465,471]	↓ [489]
E309K	↓ [465,469,471,474]	~WT [465,471]	↓ [465,471]	↓ [473,474]
D598N	↓ [465,471]	↓ (minor) [465,471]	~WT (TAX-1) [471] ↓ (axonin-1) [465]	↓ [469,473]
G698R	↓ [471]	↓ [471]	↓ [471]	Not tested
V752M	↓ [471]	↓ [471]	↓ [471]	Not tested
V768F	↓ [471]	↓ [471]	↓ [471]	Not tested
Y784C	↓ [471]	↓ [471]	↓ [471]	Not tested
P941L	↓ in CHO [471] ~WT in PC12 [474]	↓ [471]	~WT [471]	↓ [474]
R1166X	~WT [476]	Not tested (ED intact)	Not tested (ED intact)	Not tested
S1194L	~WT [469,470]	Not tested (ED intact)	Not tested (ED intact)	~WT [469]
S1224L	~WT [470,476]	Not tested (ED intact)	Not tested (ED intact)	Not tested
Y1229H	~WT [470]	Not tested (ED intact)	Not tested (ED intact)	Not tested

The effects of mutant L1CAM on cell surface delivery, homophilic/heterophilic adhesion, and neurite outgrowth are described as similar to wildtype L1CAM (~WT), upregulated (↑), or downregulated (↓).

## 8. Fetal Alcohol Spectrum Disorders and IgSF CAMs

Impaired L1CAM function has been proposed as a mechanism contributing to the pathogenesis of fetal alcohol spectrum disorders (FASDs), a leading preventable cause of intellectual and neurodevelopmental disability [490]. FASDs are characterized by mental retardation, corpus callosum agenesis, hydrocephalus and cerebellar abnormalities [491,492], overlapping with phenotypes of L1 syndrome. Several studies have shown that alcohol inhibits L1CAM-mediated cell–cell adhesion [493,494], neurite outgrowth [495] and signaling required for neuronal differentiation and survival [496,497]. Low concentrations of ethanol, equivalent to one or two maternal drinks, inhibited L1CAM-mediated adhesion in several cell lines, and this inhibition was reproduced more potently by 1-propanol and 1-butanol [493,494]. The inhibitory effects of ethanol on adhesion [498] and axon outgrowth [499] are not observed in some L1CAM-expressing cell types, indicating that L1CAM sensitivity to ethanol depends on cellular context. Prenatal exposure to ethanol in rats produces hydrocephalus-associated abnormalities without changing total L1CAM protein expression [500], suggesting that ethanol impairs L1CAM function rather than simply reducing its levels. Additionally, moderate prenatal ethanol exposure increases the total length of L1CAM-positive axons in mouse embryos, indicating altered axon growth or guidance [501].

An alcohol binding pocket has been identified at the interface between the extracellular Ig1 and Ig4 domains of L1CAM [502]. Ethanol binding is proposed to disrupt the interaction between Glu-33 in Ig1 and Tyr-418 in Ig4, destabilizing the horseshoe conformation that supports L1CAM-mediated adhesion [475,503]. This pocket also lies close to residues Leu-120 and Gly-121, at which missense mutations cause L1 syndrome [464,471,502]. L1CAM sensitivity to ethanol is also regulated by phosphorylation of the cytoplasmic domain of L1CAM [504,505]. Phosphorylation of residues S1152, Y1176, S1181 and S1248 promotes L1CAM coupling to ankyrin G and the spectrin–actin cytoskeleton, which is proposed to induce an ethanol-sensitive extracellular conformation [504].

L1CAM is also associated with lipid rafts and promotes axon growth in a raft-dependent manner [506]. In cerebellar granule neurons (CGNs), ethanol and other short-chain 1-alcohols induce translocation of L1CAM to lipid rafts, while Src is redistributed out of lipid rafts [507]. Since L1CAM-mediated neurite outgrowth requires downstream activation of Src signaling [508], this spatial separation may reduce L1CAM-dependent signaling and neurite outgrowth. Lipid raft disruption reduces both L1CAM-mediated neurite outgrowth and L1CAM sensitivity to ethanol, suggesting that ethanol may interfere with L1CAM trafficking or signaling within raft domains [507]. However, in fibroblasts, lipid raft disruption prevents ethanol-induced redistribution of L1CAM to rafts without affecting ethanol inhibition of L1CAM-mediated adhesion [509]. Together, these data suggest that raft-dependent mechanisms may be particularly important for L1CAM signaling and neurite outgrowth in neurons.

Several antagonists prevent ethanol inhibition of L1CAM as well as ethanol teratogenesis in mouse embryos [510,511,512]. These include NAPVSIPQ (NAP) and SALLRSIPA (SAL) which are active peptide fragments of two neuroprotective proteins: activity-dependent neuroprotective protein (ADNP) and activity-dependent neurotrophic factor (ADNF), respectively. Both NAP and SAL potently antagonize ethanol inhibition of L1CAM-mediated adhesion, with only NAP antagonism being irreversible [510]. NAP antagonism of ethanol inhibition of L1CAM-mediated adhesion is sufficient for preventing ethanol-induced embryotoxicity [511]. Longer-chain 1-alcohols can also act as antagonists of ethanol inhibition [513]. In cell models, the effects of ethanol and 1-butanol on L1CAM-mediated adhesion are antagonized by 1-pentanol and 1-octanol [513]. Ethanol teratogenicity is also prevented by 1-octanol antagonism [512]. Choline supplementation also reduces the effects of ethanol on L1CAM signaling, lipid raft distribution and L1CAM-mediated neurite outgrowth; however, it does not completely restore L1CAM function [514]. Overall, these findings implicate L1CAM dysfunction in prenatal ethanol toxicity and support further investigation of L1CAM as a potential therapeutic target for FASDs.

## 9. Conclusions

**Figure 3:**
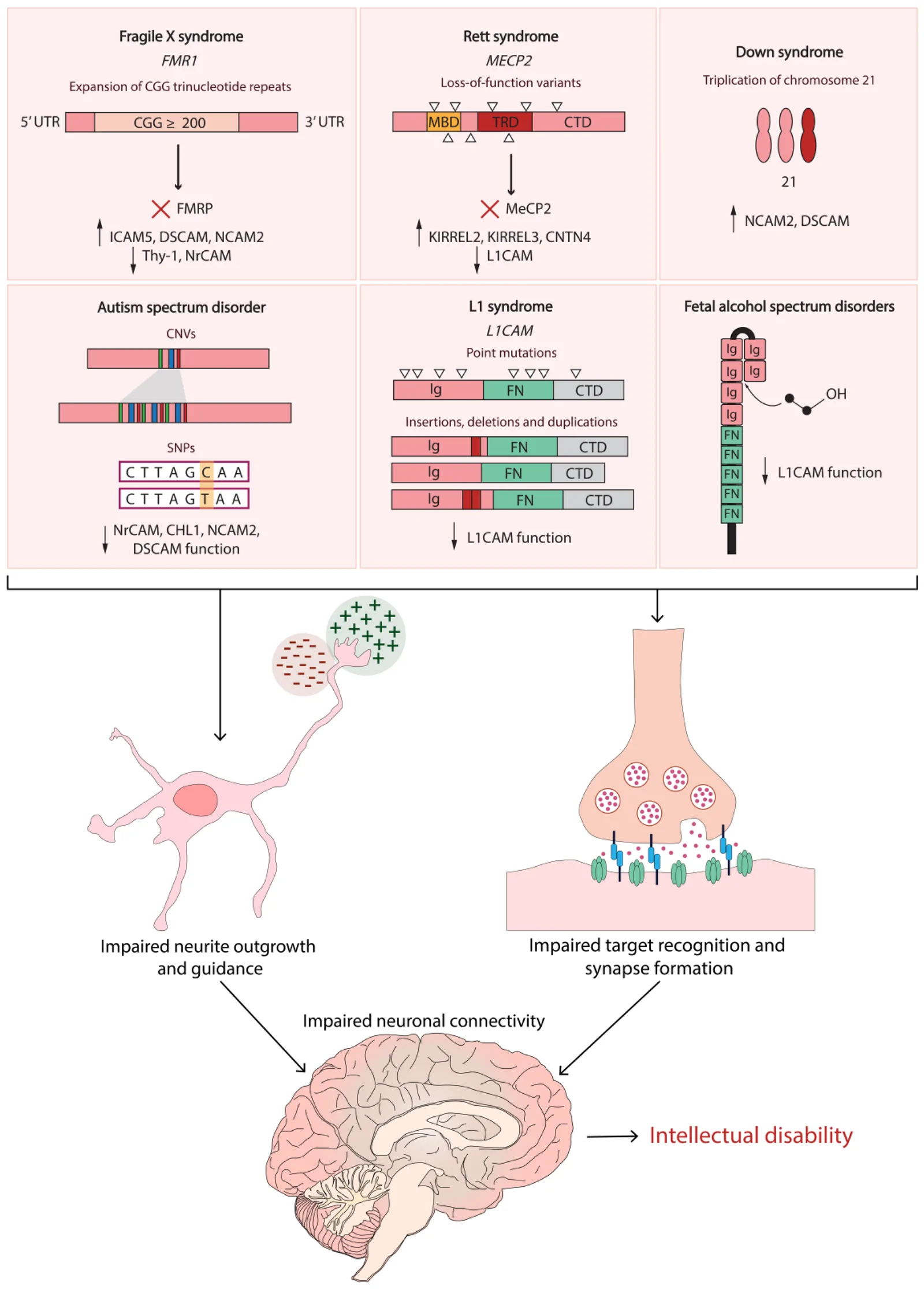
Graphical overview of the syndromes and disorders that IgSF CAMs are involved in. The levels and functions of IgSF CAMs can be affected through various mechanisms, including alterations in protein translation in Fragile X syndrome, changes in the transcription of the respective genes in Rett syndrome, triplication of chromosome 21 in DS, or genetic variants in the respective genes in ASD and L1 syndrome. In addition, environmental factors, such as prenatal alcohol exposure in FASDs, can affect the functions of L1CAM. Changes in the levels and functions of IgSF CAMs can disrupt key neurodevelopmental processes including neurite outgrowth and guidance, target recognition and synapse formation. Defects in these processes can result in impaired neuronal connectivity and contribute to the development of intellectual disability.

Accumulating evidence indicates that IgSF CAMs are involved in the pathogenesis of ID in the context of various syndromes. The levels and functions of these proteins can be affected via various mechanisms including, for example, changes in the transcription of respective genes in Rett syndrome, altered protein translation in FXS, chromosome triplication in DS, or genetic variations in respective genes in ASD and L1 syndrome (Figure 3). The functions of IgSF CAMs can also be affected by environmental factors leading to ID, such as alcohol exposure leading to FASDs (Figure 3).

Changes in levels and functions of IgSF CAMs are likely to represent only one of the factors contributing to the ID pathogenesis in these disorders. However, the significance of such changes for normal brain development is strongly supported by studies showing that deletions, duplications or mutations affecting only the genes coding for IgSF CAMs are sufficient to cause more rare cases of ID. The role of IgSF CAMs in ID is also supported by numerous studies in cellular and animal models directly showing that the loss or overexpression of IgSF CAMs affects neuronal development and synapse function and leads to behavioral changes resembling those found in humans with ID. Together, these studies indicate that IgSF CAMs need to be considered as important therapeutic targets in ID, and their functions need to be assessed as mechanistic readouts to determine the effectiveness of new ID treatments that are yet to be developed.

## Data Availability

No new data were created or analyzed in this study. Data sharing is not applicable to this article.

## Abbreviations

The following abbreviations are used in this manuscript:

21q	Long arm of chromosome 21
A2BP1	Ataxin 2-binding protein 1
Aβ	Amyloid-β
Abl	Abelson
AD	Alzheimer’s disease
ADNF	Activity-dependent neurotrophic factor
ADNP	Activity-dependent neuroprotective protein
Akt	Protein kinase B
APP	Amyloid precursor protein
ASD	Autism spectrum disorder
BACE1	β-site amyloid precursor protein cleaving enzyme 1
BDNF	Brain-derived neurotrophic factor
C4da	Class IV dendritic arborization
CAM	Cell adhesion molecule
CGN	Cerebellar granule neuron
ChIP-seq	Chromatin immunoprecipitation sequencing
CHL1	Close homolog of L1
CLSTN1	Calsyntenin-1
CNTN4	Contactin 4
CNTN6	Contactin 6
CNV	Copy number variation
CREB1	cAMP-responsive element-binding protein 1
CRISPR-Cas9	Clustered regularly interspaced short palindromic repeats–CRISPR-associated protein 9
c-Src	Cellular sarcoma
DS	Down syndrome
DSCAM	Down syndrome cell adhesion molecule
ED	Extracellular domain
E/I	Excitatory/inhibitory
ER	Endoplasmic reticulum
ERK1/2	Extracellular signal-regulated kinase 1 and 2
FASDs	Fetal alcohol spectrum disorders
FMR1	Fragile X messenger ribonucleoprotein 1
FMRP	Fragile X messenger ribonucleoprotein
FNIII	Fibronectin type III
FXS	Fragile X syndrome
GABA	γ-Aminobutyric acid
GDD	Global developmental delay
GluA2	Glutamate ionotropic receptor AMPA type subunit 2
GluN2A	Glutamate ionotropic receptor NMDA type subunit 2A
GluN2B	Glutamate ionotropic receptor NMDA type subunit 2B
GPI	Glycosylphosphatidylinositol
hPSC	Human pluripotent stem cell
HSA21	Human chromosome 21
HSAS	Hydrocephalus with stenosis of the aqueduct of Sylvius
ICAM5	Intercellular adhesion molecule 5
ID	Intellectual disability
Ig	Immunoglobulin
IGF1	Insulin-like growth factor 1
IgLON	Immunoglobulin superfamily containing LAMP, OBCAM and neurotrimin
IgSF	Immunoglobulin superfamily
iPSC	Induced pluripotent stem cell
iTRAQ	Isobaric tags for relative and absolute quantitation
IQ	Intelligence quotient
KH	K homology
KIRREL2	Kirre-like nephrin family adhesion molecule 2
KIRREL3	Kirre-like nephrin family adhesion molecule 3
L1CAM	L1 cell adhesion molecule
LTD	Long-term depression
LTP	Long-term potentiation
m^6^A	N^6^-methyladenosine
MADS	MCM1, agamous, deficiens, and serum response factor
MAP1B	Microtubule associated protein 1B
MASA	Mental retardation, aphasia, shuffling gait, adducted thumbs
MeCP2	Methyl-CpG binding protein 2
Mef2c	Myocyte enhancer factor 2C
mGluR5	Metabotropic glutamate receptor 5
mGluR-LTD	Metabotropic glutamate receptor-dependent long-term depression
miRNA	microRNA
mTOR	Mechanistic target of rapamycin
mTORC	Mechanistic target of rapamycin complex 1
NAP	NAPVSIPQ
NCAM	Neural cell adhesion molecule
NCAM2	Neural cell adhesion molecule 2
NCoR	Nuclear receptor co-repressor
NEGR1	Neuronal growth regulator 1
Nfasc	Neurofascin
NGS	Next-generation sequencing
NMDA	N-methyl-D-aspartate
NPC	Neural precursor cell
NrCAM	Neuronal cell adhesion molecule
PI3K	Phosphoinositide 3-kinase
PIGQ	Phosphatidylinositol glycan biosynthesis class Q
pLI	Probability of loss-of-function intolerance
PSD-95	Postsynaptic density protein-95
Rab11	Ras-related protein in brain 11
RGC	Retinal ganglion cell
RISC	RNA-induced silencing complex
S6K1	Ribosomal protein S6 kinase 1
shRNA	Short hairpin RNA
SNP	Single-nucleotide polymorphism
snRNA-seq	Single-nucleus RNA sequencing
SNV	Single-nucleotide variant
Src	Sarcoma
SynCAM	Synaptic cell adhesion molecule
TAX-1	Transient axonal glycoprotein 1
Thy-1	Thy-1 membrane glycoprotein
UNC5C	Uncoordinated-5C
VGAT	Vesicular GABA transporter
VGLUT	Vesicular glutamate transporter
Xq	X chromosome
